# Dysregulated 3′-end processing of 18S pre-rRNA decreases mtPNPase efficiency in plant mitochondria

**DOI:** 10.1093/nar/gkag609

**Published:** 2026-06-17

**Authors:** Malgorzata Kwasniak-Owczarek, Blazej Przystajko, Artur Tomal, Huy Cuong Tran, Urszula Kazmierczak, Holger Eubel, Noah Ditz, Olivier Van Aken, Hanna Janska

**Affiliations:** Department of Cellular Molecular Biology, Faculty of Biotechnology, University of Wroclaw, F. Joliot-Curie 14A, 50-383 Wroclaw, Poland; Department of Cellular Molecular Biology, Faculty of Biotechnology, University of Wroclaw, F. Joliot-Curie 14A, 50-383 Wroclaw, Poland; Department of Cellular Molecular Biology, Faculty of Biotechnology, University of Wroclaw, F. Joliot-Curie 14A, 50-383 Wroclaw, Poland; Department of Biology, Lund University, Lund 221 00, Sweden; Department of Cellular Molecular Biology, Faculty of Biotechnology, University of Wroclaw, F. Joliot-Curie 14A, 50-383 Wroclaw, Poland; Institute of Plant Genetics, Leibniz University Hannover, Herrenhäuser Str. 2, Hannover 30419, Germany; Institute of Plant Genetics, Leibniz University Hannover, Herrenhäuser Str. 2, Hannover 30419, Germany; Department of Biology, Lund University, Lund 221 00, Sweden; Department of Cellular Molecular Biology, Faculty of Biotechnology, University of Wroclaw, F. Joliot-Curie 14A, 50-383 Wroclaw, Poland

## Abstract

In plant mitochondria, mitochondrial polynucleotide phosphorylase (mtPNPase) is a key 3′→5′ exoribonuclease. Here, we describe the accumulation of mtPNPase substrates in Arabidopsis mutants, such as *rps10* (deficient in the mitoribosomal protein uS10m), *mtran1-2/2-2* (lacking the mitoribosomal proteins mTRAN1 and mTRAN2), and *rpoTmp* (deficient in plastid- and mitochondrial-targeted RNA polymerase). This accumulation is not due to a reduced mtPNPase expression; instead, all three mutants exhibit perturbations in mitoribosome biogenesis associated with inefficient mtPNPase-dependent 3′-end processing of 18S pre-ribosomal RNA. We propose a spatial sequestration model in which mtPNPase becomes trapped by incompletely matured 18S precursors, limiting its availability for other substrates. In addition, the *rps10* mutant displays a partial shift of both mtPNPase and mitoribosomes from membrane-associated to soluble fractions, suggesting a mutant-specific alteration that may further modulate mtPNPase function. Together, these findings demonstrate that proper mitochondrial small-subunit (mtSSU) biogenesis is essential for effective mtPNPase function and balanced mitochondrial RNA metabolism. Thus, mitoribosomes act not only as translation machines but also as regulators of mitochondrial RNA homeostasis, linking ribosome biogenesis to the composition and turnover of the mitochondrial transcriptome.

## Graphical Abstract

**Graphical Abstract:**
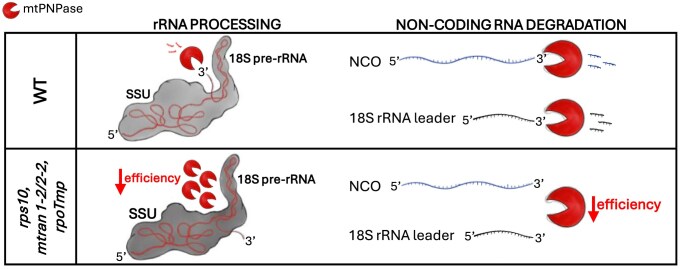

## Introduction

RNA metabolism in plant mitochondria differs significantly from that of yeast and animal mitochondria [1]. Given the relaxed nature of transcription in plant mitochondria, RNA maturation is believed to play a dominant role in shaping the mitochondrial transcriptome; the gene product abundance is also controlled at the translational stage [2, 3]. However, very little is known about the connections between RNA maturation and the translational machinery in plant mitochondria. Only a few post-transcriptional processing proteins, such as RNA editing factors [4], Protein-Only RNase P (PRORP1) [5], and Putative Mitochondrial Helicase (PMH) [6] have been reported to associate with mitoribosomes. This is in sharp contrast to yeast and animal mitochondria where such factors are in close spatial relationship with ribosomes via the creation of structures such as MIOREX (Mitochondrial Organization of Gene Expression) in yeast [7] and RNA granules in mammals [8].

One of the key enzymes of RNA metabolism in bacteria, as well as chloroplasts and mitochondria is the 3′–5′ exoribonuclease polynucleotide phosphorylase (PNPase) [9]. The importance of PNPase in plant mitochondria [mitochondrial polynucleotide phosphorylase (mtPNPase)] is evidenced by the fact that null mutants are embryo-lethal [10]. A downregulation of mtPNPase in Arabidopsis results in the accumulation of several classes of RNAs: messenger RNA (mRNA) and ribosomal RNA (rRNA) precursors containing long 3′ extensions, rRNA and transfer RNA maturation by-products, chimeric open reading frames (ORFs), antisense RNA, and intergenic transcripts [11]. In all organisms, PNPase functions as an RNA-degrading enzyme but additional functions have been identified in some organisms. In bacteria and chloroplasts, PNPase also exhibits poly(A) polymerase activity [12, 13]. Human mitochondrial PNPase (hPNPase) was unexpectedly discovered in the intermembrane space of mitochondria as a peripheral membrane protein, where it functions as an RNA-importing enzyme [14]. The crystal structures of bacterial and human PNPases reveal a ring-shaped homotrimeric architecture, with the central channel comprising the site of catalytic activity [9]. *In vivo*, PNPases form trimers or dimers of trimers or are part of multiprotein complexes. Such PNPase complexes have been described for bacteria [15] and human mitochondria [16, 17], chloroplastic PNPase occurs only as a homo-multimer [18]. Nothing is known so far about the nature of the complexes/assemblies formed by mtPNPase in plant mitochondria.

Plant mitoribosomes are among the largest and most complex ribosomes. It has recently been shown that their small subunit (mtSSU) is actually very large, larger than the large subunit (mtLSU) [4, 19]. Compared with bacterial ribosomes, the plant mitoribosomes are richer in proteins and their rRNAs have large expansion segments. Recently, using different experimental approaches, two groups [4, 19] independently reported, respectively, 92 and 94 proteins, as components of the *Arabidopsis thaliana* mitoribosome, compared with only 54 proteins of the *Escherichia coli* ribosome. The two datasets had 74 proteins in common and showed the presence of nonconventional proteins, most belonging to the PPR class, in both subunits. It is noteworthy that the composition of plant mitoribosomes could be heterogeneous due to the presence of paralogous genes encoding mitoribosomal proteins [20]. It should also be highlighted that at least a fraction of plant mitoribosomes is tightly bound to the inner mitochondrial membrane [21]. However, so far, the mode of such attachment has not been determined. Since the Arabidopsis genome does not encode homologs of mammalian ml45, yeast Mba1, or Chlamydomonas ml119, which are required in these organisms to tether the mitoribosome to the Oxa1 insertase in the mitochondrial membrane, it is conceivable that in land plants the mitoribosomes use a unique mode of membrane attachment [22].

A heterogeneous population of mitoribosomes has been observed in Arabidopsis mutants generated by silencing the nuclear gene *RPS10* encoding uS10m, an SSU mitochondrial ribosomal protein [3]. In the *rps10* mutant partially assembled SSUs coexisted with WT ones and an excess of free mtLSUs. Ribosome profiling revealed that *rps10* mitoribosomes protect transcript fragments that are shorter than those protected by WT mitoribosomes, likely reflecting the structural distortion of the mtSSU [2]. The altered structure of *rps10* mitoribosomes also caused a weaker maintenance of the correct open reading frame as well as a markedly more efficient translation of ribosomal proteins, MatR, and TatC proteins.

Here we extend the above studies and show that the dysregulation of mtSSU biogenesis due to a decreased abundance of mitoribosome protein uS10m, or a deficiency of another mitoribosomal protein mTRAN, or of the dual-targeted mitochondrial and plastid RNA polymerase, affects the functionality of mtPNPase in shaping the mature transcriptome in plant mitochondria.

## Materials and methods

### Plant material and growth conditions

Transgenic *A. thaliana* plants with RNA interference (RNAi)-mediated silencing of the *RPS10* gene expression [further referred to as *rps10* (P2 and P3 phenotypes)] [23] were the main plant material used in this study. In some experiments several T-DNA insertional mutants were also investigated: *ftsh4-1* (SALK_035 107) [24], *rpoTmp-1* (GABI_286E07) and *rpoTmp-2* (SALK_132 842) [25], *rrd1* [26], *ahg2-1* [27], *mtran1-1/2–1* and *mtran1-2/2–2* [28], *amiR-PNP-3* [29]. All the mutants were in *Columbia* (Col-0) background with the exception of *rrd1*, which was in *Landsberg erecta* (Ler) background. For the *rps10, amiR-PNP-3*, and *rpoTmp* mutants, experiments were performed on the youngest altered leaves from plants growing in the soil in conditions described previously in [3, 29], and [25], respectively. For all the other mutants, young seedlings were used grown exactly as described previously: *ftsh4-1* (2-week-old seedlings) [30], *rrd1* (9-day-old seedlings) [31], *ahg2-1* (3-week-old seedlings) [32], *mtran1-1/2–1* (11-day-old seedlings), and *mtran1-2/2–2* (18-day-old seedlings) [28].

### Isolation of mitochondria

Mitochondria were isolated from the youngest leaves or whole seedlings exactly as described in [33]. Mitochondrial fractions were frozen in liquid nitrogen and stored at −80°C or used directly in experiments.

### Isolation of RNA

RNA was isolated directly from the youngest leaves or whole seedlings (total RNA), as well as from the mitochondrial fractions (mitochondrial RNA). In both cases the isolation was performed using the GeneMATRIX Universal RNA purification kit (EURx) with an additional step of DNase I (RNase-free) (Ambion, Thermo Fisher Scientific) treatment.

### Fractionation of RNA in sucrose gradients

RNA was isolated from fractionated homogenized leaf tissue as described previously by [3]. Wild-type (WT) or *rps10* leaves (200 mg fresh weight) were ground in 1 ml of extraction buffer [200 mM Tris–HCl, pH 9.0; 200 mM KCl; 35 mM MgCl_2_; 25 mM ethylene glycol‐bis(β‐aminoethyl ether) N,N,N′,N′‐tetraacetic acid (EGTA); 200 mM sucrose (Suc); 100 mM β-mercapthoethanol; 1% (v/v) Triton X-100; 2% (v/v) polyoxyethylene-10-tridecyl ether; 6% (w/v) digitonin; 1 mg/ml heparin; 100 μg/ml chloramphenicol; and 25 μg/ml cycloheximide] and centrifuged at 4°C and 13 200 × *g* for 5 min. The cleared extract was supplemented with 1/20 volume of 10% (w/v) sodium deoxycholate and fractionated by ultracentrifugation (4°C; 200 000 × *g*; 80 min) on a Suc density step gradient [56:40:30:15% (w/v) Suc in 40 mM Tris–HCl, pH 8.5; 20 mM KCl; 10 mM MgCl_2_; 100 μg/ml chloramphenicol; and 500 μg/ml heparin] into 10 fractions of equal volume. To discriminate between nonpolysomal RNA (fractions 1–5) and polysomal RNA (fractions 6–10), ribosomes were dissociated by adding 1.2 mM of puromycin to the extract before loading it on the Suc gradient. The fractions were subjected to RNA extraction with phenol/chloroform/isoamyl alcohol (25:24:1), and the RNA was precipitated with 2.5 M LiCl to remove heparin. Purified RNA was used for reverse transcription quantitative polymerase chain reaction (RT-qPCR) as described below.

### RT-qPCR analysis

Reverse transcription was performed using up to 2 μg of RNA, random hexamers and High-Capacity complementary DNA (cDNA) Reverse Transcription Kit (Thermo Fisher Scientific) according to the manufacturer’s directions. For reverse transcription of mitochondrial RNA, External Standard Kit (Lambda PolyA) (TaKaRa Bio) was added according to manufacturer’s manual. The resulting cDNA was used as a template for quantitative real-time PCR analysis on a Bio-Rad CFX96 Touch Real-Time PCR Detection System with dedicated software (Bio-Rad) and with sets of primers described in Supplementary Table S1. The content of individual transcripts in mitochondrial RNA was determined using λ polyA + RNA-A as a reference while for total RNA the *ACT2* gene (At3g18780) was used as a reference.

### Northern blot analysis

One microgram of mitochondrial RNA was resolved in denaturing conditions in 1.2% [analysis of internal transcribed region (ITS) and noncoding overexpressed (NCO)] or 1.5% (analysis of 5S rRNA) agarose-formaldehyde gels. Blotting and hybridization were performed as described in [34] with slight modifications. The following oligonucleotide probes biotinylated on the 5′ terminus were used: ITS 5′ [Btn] CTTGGTTGGGGGAAGAGGAACGAAGTCCATCGCGAA 3’, 5S rRNA 5’ [Btn] TCACCGGGCTTGGACCATGTCTCC CGAACAATCTCAGTACATATGGCGCAAGACGATTCCA CATATCGAGGTCGGAATGGGATCGGGTGTTTTCACG TCTCACCGTAGTGCCCGGTTT 3’ and NCO 5’ [Btn] CAAAAGAAATGAAATTTACGTGATGCGGGAACCTAC TTTCCCTAG 3’. Hybridization was performed overnight in PerfectHyb Plus buffer (Sigma–Aldrich) at 50°C and then the membrane was washed twice with Wash Buffer I [1 × saline-sodium citrate (SSC), 0.2% (w/v) sodium dodecyl sulphate (SDS)] and twice with Wash Buffer II (0.1× SSC, 0.2% SDS) for 10 min at 50°C. The biotin-labeled probes were detected using the Chemiluminescent Nucleic Acid Detection Module Kit (Thermo Fisher Scientific) according to the manufacturer’s directions. Data were analyzed using G:Box software (Syngene).

### Circular RT-PCR

Circular RT-PCR (cRT-PCR) was used to determine the termini of 18S, 26S, and 5S rRNA precursors. Mitochondrial RNA (5 µg) was circularized with 30 U of T4 RNA ligase (New England Biolabs), according to the manufacturer’s instructions. After ethanol precipitation, the circularized RNA served as template for cDNA, which was synthesized using a First-Strand cDNA Synthesis kit with SuperScript™ II reverse transcriptase (Invitrogen). Polymerase chain reaction (PCR) amplification was performed with gene specific primers flanking the ligation junction (forward primer near the 3′ end and reverse primer near the 5′end). Primers sequences for reverse transcription and PCR amplification are listed in Supplementary Table S1. PCR conditions were: 35 cycles of 30 s at 95°C; 30 s at 52°C; and 1 min at 72°C. PCR products were separated in 1% agarose gel and bands corresponding to rRNA precursors were excised, purified using Agarose-Out Purification kit (EurX)/GeneJET Gel Extraction Kit (Thermo Scientific), cloned using the pGEM-T Easy Vector System (Promega)/CloneJET PCR Cloning Kit (Thermo Scientific), and sequenced. The obtained sequences were analysed using SnapGene software.

### Sodium dodecyl sulfate–polyacrylamide gel electrophoresis and immunoblotting

Sodium dodecyl sulfate–polyacrylamide gel electrophoresis (SDS–PAGE) was performed on 8% or 10% polyacrylamide gels according to [35], with equal amounts of mitochondrial proteins per lane, as indicated in individual figures. For immunoblotting, proteins were transferred onto polyvinylidene difluoride (PVDF) membrane (Bio-Rad) and probed with the following primary rabbit antibodies: against the Arabidopsis mitoribosomal protein, uS14m (AS16 4094, Agrisera), uS10m (AS15 3067, Agrisera), uS4m (AS15 3068, Agrisera), and uL16m (AS15 3069, Agrisera), as well as against mtPNPase (antibodies were obtained, respectively, from D. Gagliardi, University of Strasbourg, France, against a protein corresponding to amino acids 659–991 of AtmtPNPase expressed in *E. coli*; or from PhytoAb, against the peptide, EKAGKQKKEYKLSMLSD corresponding to amino acids 271–287 of AtmtPNPase), LETM1 (antibody obtained from O. Van Aken, Department of Biology, Lund, Sweden), and isocitrate dehydrogenase (IDH; AS06 203A, Agrisera). Goat anti-rabbit antibodies conjugated with horseradish peroxidase were used as secondary antibodies (Agrisera, AS09 602). The proteins of interest were visualized by chemiluminescence with the WesternBlot Quantum HRP substrate (Advansta) and a GBox imager (Syngene).

### Label-free quantitative shotgun mass spectrometry

Preparation of mitochondrial fractions from WT, P2, and P3 mutants for label-free quantitative shotgun mass spectrometry was conducted following the single-pot-solid-phase-enhanced sample preparation (SP3) protocol [36] adjusted to *A. thaliana* plant material [37] and as outlined in [38]. Resuspended mitochondria (5 µl, equaling 50 µg protein) were supplemented with 15 µl ultra-pure water and subsequently mixed with 20 µl of 2× SDT buffer [8% (w/v) sodium dodecyl sulfate; 0.2 M dithiothreitol (DTT); 0.2 M Tris–HCl, pH 7.6] followed by incubation for 1 h at 60°C and 1000 rpm in a thermoshaker (TS-100, Kisker Biotech, Steinfurt, Germany). Undissolved material was removed by centrifugation for 10 min at 20 000 × *g*. Then, 30 µl of the supernatant (containing solubilized proteins) were supplemented with 100 mM iodoacetamide to yield a final concentration of 20 mM and incubated for 30 min and 600 rpm in the dark. The reaction was stopped by adding 100 mM DTT to a final concentration of 5 mM.

Sera-Mag carboxylate-modified beads hydrophilic solids were mixed 1:1 with hydrophobic solids (both Cytiva, Freiburg, Germany), yielding a bead concentration of 20 µg µl^−1^. Each sample was supplemented with 600 µg of beads and protein binding was induced by addition of pure ethanol to yield a final ethanol concentration of 50% (v/v). After incubation for 10 min and 1000 rpm, beads were pelleted for 2 min in a magnetic rack and washed three times in 140 µl of 80% (v/v) ethanol. Washed beads were transferred into low-binding tubes (Low Binding Micro Tubes, Sarstedt, Germany), and 3.4 µg of sequencing-grade modified Trypsin (V5111, Promega) dissolved in 60 µl 50 mM ammonium bicarbonate was added to each sample. Digestion was performed at 37°C and 1000 rpm overnight. After addition of 1% (v/v) formic acid (FA) to abolish trypsin activity, beads were pelleted for 2 min on a magnetic rack and the peptide-containing supernatant was transferred to a new low-binding tube. Beads were mixed again with 60 µl of fresh 50 mM ammonium bicarbonate. After incubation for 2 min at room temperature and subsequent pelleting the beads in a magnetic rack, this supernatant was pooled with the previous supernatant. After drying of the sample in a vacuum centrifuge and dissolving the analyte molecules in 100 µl 0.1% (v/v) FA, peptides were desalted on 50 mg Sep-Pak tC18 columns (WAT054960, Waters) and quantified using the Pierce Quantitative Colorimetric Peptide Assay Kit (Thermo Fischer Scientific, Dreieich, Germany) according to the manufacturer’s instructions. Subsequently, peptides were first dried in a vacuum centrifuge again and resuspended in 0.1% (v/v) FA to yield a concentration of 200 ng/µl, of which 1 µl was injected into a nanoElute HPLC coupled to a timsTOF Pro ion mobility spectrometry quadrupole time of flight mass spectrometer (both Bruker, Bremen, Germany). Peptide separation as well as MS/MS were performed as outlined in [38].

Raw data were queried against an in-house Arabidopsis database using MaxQuant (ver 2.0.3.0) [39]. Label-free quantification (LFQ) values were then used for a quantitative comparison of P2 and P3 *rps10* mutant mitochondria against WT mitochondria using the Perseus software package (ver 2.0.3.0) [40]. Protein groups were classified as ‘quantifiable’ if LFQ-values were available for at least two replicates within each group. Missing values were imputed separately for each column using pre-set Perseus parameters (width, 0.3; down shift, 1.8) Two-sample *t*-test with permutation-based false discovery rate (FDR) was performed using a significance threshold of 0.01 (S0, 0.2). The intracellular localization of proteins was annotated using the SUBAcon algorithm [41] and the functional context was created using MapMan annotation [42].

### Sodium carbonate and freeze–thaw fractionation of mitochondria

Freshly isolated mitochondria from WT and *rps10* plants were fractionated using a sodium carbonate treatment as described in [43] with slight modifications. Mitochondrial fractions (50 μg of protein) were resuspended in 100 μl of 100 mM sodium carbonate at pH values of 11.0, 11.5, or 12.5, and incubated for 30 min on ice. Then, mitochondrial membranes were pelleted for 30 min at 21 460 × *g* and 4°C and considered as the membrane fraction. Proteins from the supernatant were precipitated by adding 0.1 vol of 100% trichloroacetic acid (TCA) and incubation at −20°C for 1 h, followed by pelleting for 15 min at 21 460 × *g* and 4°C. The pellet was washed with 100 μl of ice-cold 80% (v/v) acetone, centrifuged for 5 min as above, and considered as the soluble fraction. Both membrane and soluble fractions were resuspended in 20 μl of 2× Laemmli Sample buffer (Bio-Rad), boiled for 10 min and submitted to SDS–PAGE. To aid the solubilization of TCA-precipitated proteins, the “soluble fraction” was subjected to sonication (10 min, 50°C) in 2× sample buffer prior to heating. Separated proteins were transferred onto PVDF membrane and subjected to immunoblotting,

Freeze–thaw fractionation of mitochondria was performed according to [44] with some modifications. In brief, mitochondrial fractions (100 μg of protein) were resuspended in 100 µl of fractionation buffer (10 mM N-tris[hydroxymethyl]methyl-2-aminoethanesulfonic acid (TES), pH 7.0;10 mM MgCl_2_; 1 mM DTT; 40 mM KCl) and subjected to five cycles of freeze/thawing in liquid N_2_ to rupture membranes. The suspension was then incubated on ice for 30 min and centrifuged for 15 min at 18 000 × *g* and 4°C, yielding a soluble fraction in the supernatant, and a membrane fraction in the pellet. The pellet was resuspended again in 100 µl of fractionation buffer and centrifuged as above. Supernatants from both centrifugations (200 µl) were combined and the pellet was resuspended in 200 µl of fractionation buffer. Then, 3 µl of external standard [1000× diluted λ polyA + RNA-A (1.8 × 10^7^ cop/µl)] from the Lambda PolyA External Standard Kit (TakaRa Bio) was added to the obtained fractions. RNA was extracted separately from the soluble and membrane fractions and analysed.

## Results

### In addition to uS10m, six other mtSSU proteins are under-represented in the *rps10* mutant

We previously reported that silencing of *RPS10* encoding the mitochondrial ribosomal uS10m protein in a hemizygous *rps10* mutant of Arabidopsis leads to increased abundance of the mtSSU and mtLSU subunits, with some mtSSUs incompletely assembled, missing the uS10m protein [3]. Here, to determine whether the defective subunits contain all the other proteins, we performed a shotgun proteomic analysis to determine the abundance of mitoribosomal proteins in two phenotypes of *rps10* (P2 and P3) relative to the WT. P2 and P3 phenotypes exhibit a comparable decrease in the *RPS10* transcript level but differ by the timing of the onset of the silencing [23]. P2 represents an early-onset silencing during vegetative growth, whereas P3 represents a late-onset silencing occurring in 7-week-old plant [3, 23].

**Figure 1.**
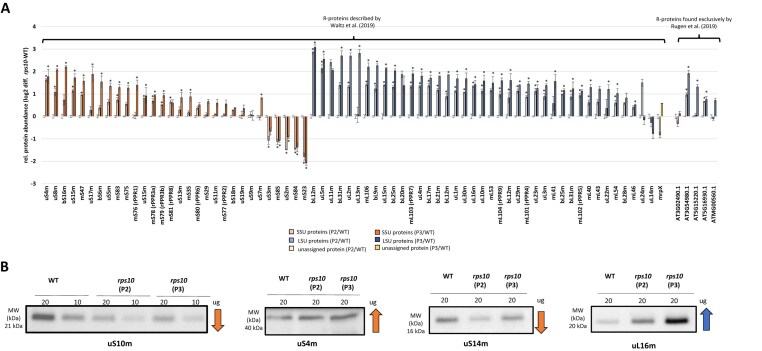
Abundance of mitoribosomal proteins in *rps10* compared with WT. (**A**) Abundance of mitoribosomal proteins in *rps10* phenotypes P2 and P3 as identified by shotgun analysis relative to WT. Log_2_ difference of average LFQ-vaules (*n* = 3) is shown. The analyzed Arabidopsis mitoribosomal components were selected based on data reported by [19] and [4]. Orange – small subunit (mtSSU) proteins, blue – large subunit (mtLSU) proteins, yellow – mitoribosomal protein unassigned to either subunit. The values are means of three biological replicates, with error bars representing standard error. Asterisks indicate statistical significance (FDR, 0.01; S_0_, 0.2). (**B**) Abundance of selected mitoribosomal proteins in WT and *rps10* determined by immunoblotting. Equal amounts (20 µg; or 20 and 10 µg) of mitochondrial protein were probed with antibodies against uS10m, uS4m, uS14m, and uL16m proteins. Representative blots are shown. A graph showing densitometric quantifications of relative protein levels is shown in Supplementary Fig. S1. Molecular weight (MW) markers are indicated in kDa.

In both P2 and P3, we could identify 31 of the 41 known mtSSU protein groups, as well as 43 protein groups of the 57 mtLSU proteins. In addition, one mitoribosomal protein (mrpX) not assigned to either subunit according to [19] was found as well (Fig. 1A and Supplementary Table S2). We also found five protein groups that were identified as Arabidopsis mitoribosomal proteins exclusively by [4] (Fig. 1A and Supplementary Table S2). With the exception of uL14m and uL24m, the remaining identified mtLSU protein groups are of increased abundance in the *rps10* mutant when compared to the WT, most of which in statistically significant manner. Most of the mtSSU protein groups were also more abundant in *rps10*, but five protein groups showed a decreased level: uS2m, uS3m, mS23, mS84, and mS85. Also here, the majority of protein groups responded in a statistically significant manner. The similar decrease in the abundance of the six mtSSU proteins between two developmentally independent phenotypes of *rps10* indicates that the effect reflects a direct consequence of uS10m loss rather than a secondary response.

The uS10m protein was not among the mitoribosomal proteins identified in these experiments because it did not meet the strict statistical conditions applied to the shotgun analysis (see the ‘Materials and methods’ section). Therefore, we determined its level by immunoblotting together with two additional components of mtSSU, uS4m and uS14m, as well as one mtLSU protein, uL16m. The abundance of uS10m and uS14m was lower, and that of uS4m and uL16m was higher in *rps10* than in the WT (Fig. 1B and Supplementary Fig. S1). Thus, for uS4m and uL16m, western blotting confirms shotgun proteomics results.

### Deficiency of uS10m affects the formation of the 3′ end, but not the 5′ end of 18S rRNA in Arabidopsis mitochondria

**Figure 2.**
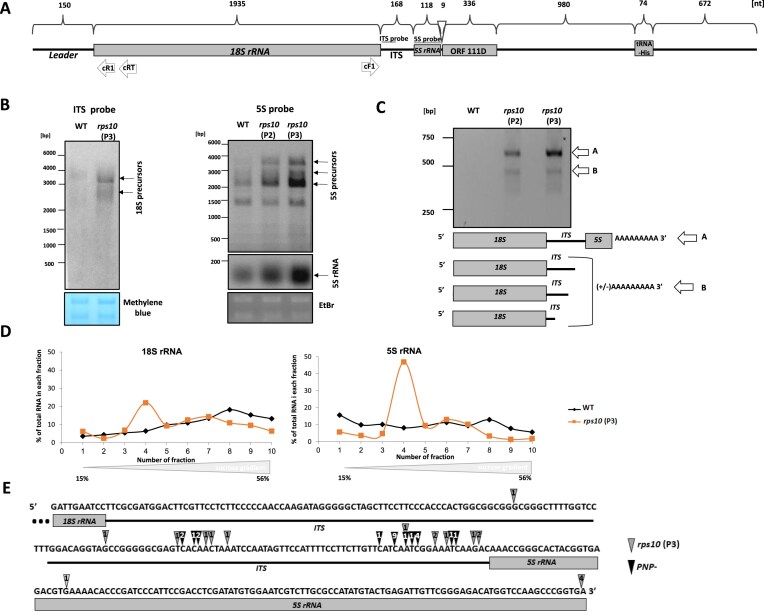
Accumulation of 18S and 5S rRNA precursors in *rps10* compared with WT. (**A**) Structure of the *Atm-15* polycistronic pre-rRNA transcript, which includes sequences of 18S and 5S rRNA. The diagram shows the organization of the *trnH-orf111d-rrn5-rrn18* gene cluster and the positions of primers used for cRT-PCR. Gray lines indicate regions targeted by probes in Northern blot analysis. Fragment sizes within *Atm-15* are given in nucleotides. (**B**) Northern blot showing increased levels of 18S and 5S rRNA precursors in *rps10*. A 5′-biotinylated oligonucleotide probe complementary to either ITS or 5S rRNA was hybridized to mitochondrial RNA. Arrows mark putative precursor transcripts; methylene blue or ethidium bromide staining serves as a loading control. (**C**) Mapping of the 5′ and 3′ termini of 18S precursors by cRT-PCR. Panel A shows the primer positions used for cDNA synthesis (cRT) and PCR amplification (cF1 and cR1). The upper image presents a negative of an ethidium bromide-stained agarose gel showing cRT-PCR products (bands A and B). The lower diagram illustrates the structure of the 18S rRNA precursors identified in *rps10*, which share the mature 5′ end but differ in their 3′ termini, based on sequencing of products from bands A and B. (**D**) Distribution of 18S and 5S rRNA in fractions separated by sucrose gradient ultracentrifugation of total RNA isolated from WT and *rps10* (P3) plants. RT-qPCR was used to determine the abundance of the two rRNA species across fractions. Graphs show the relative content of each rRNA species expressed as a percentage of its total amount. (**E**) Comparison of 3′ ends of 18S precursors in *rps10* (gray arrowheads, this work) and mtPNPase-deficient plants (black arrowheads [48]). Numbers in arrowheads indicate sequenced clones.

Since the uS10 protein in bacteria [45] and mammals [46] is known to participate in the regulation of rRNA maturation, we decided to investigate this process in *rps10*. In Arabidopsis mitochondria, the mature 18S and 5S rRNAs of mtSSU originate from a 4125 base pair (bp) polycistronic precursor transcript, Atm-15 [47]. This transcript encompasses the *trnH*-*orf111d*-*rrn5*-*rrn18* gene cluster, including intergenic spacers. The region between 18S rRNA and 5S rRNA is referred to as the ITS. Additionally, Atm-15 contains external transcribed regions at both ends, with the 5′ region known as the leader sequence (see Fig. 2A).

To detect unprocessed precursors of 18S and 5S rRNAs, we performed Northern blot analysis using labeled probes specific to the ITS region and 5S rRNA. For the ITS probe, which detects 18S rRNA intermediates, two distinct signals were observed, both stronger in *rps10* than in WT, indicating impaired processing. The 5S probe revealed increased intensity of three long precursor forms in *rps10*, consistent with accumulation of unprocessed 5S rRNA species (Fig. 2B). Since 18S rRNA maturation in plant mitochondria involves a complex, multistep pathway, with current knowledge mostly limited to its late stages [48], we focused subsequent analyses on the processing of the 5′ and 3′ ends of the 18S rRNA.

To map the 5′ and 3′ termini of the18S precursors we performed cRT-PCR using primer cRT for cDNA synthesis and primers cF1 and cR1 for PCR amplification (Fig. 2A and C). Two distinct bands of ∼600 bp and ∼450 bp were visible after electrophoresis of PCR products for *rps10* but not for the WT (Fig. 2C). The PCR products from the 600 bp band were all identical in size, while the products from the ∼450 bp band showed some size heterogeneity (Supplementary Fig. S2). Importantly, sequencing showed that all the PCR products had the same 5′ end, identical with that of mature 18S rRNA but different 3′ ends (Fig. 2C and Supplementary Table S3). The 600 bp PCR products included a segment of 18S rRNA, the complete ITS region, and the full 5S rRNA, along with a polyadenylated 3′ end. In contrast, the PCR products from ∼450 bp band contained a portion of 18S rRNA and truncated ITS sequences ranging in length from 74 to 169 bp (Supplementary Table S3). Five out of the 14 precursors from the ∼450 bp band were polyadenylated.

To confirm these observations, we used quantitative RT-PCR to determine the distribution of the 18S and 5S rRNA sequences in *rps10* (P3) and WT total RNA sample following their separation by sucrose density gradient centrifugation (Fig. 2D). At least some of the 18S and 5S rRNAs co-sediment in the same nonpolysomal fraction in *rps10*, but not in the WT. This result confirms that incompletely processed precursors containing 18S rRNA fused to 5S rRNA accumulate in *rps10*. Taken together, our findings indicate that in *rps10* the 18S precursors are processed correctly at the 5′ end but not at the 3′ end. Interestingly, Arabidopsis plants lacking mtPNPase (PNP-plants) had been reported to exhibit a similar defect in 18S rRNA maturation [48]. As shown in Fig. 2E, the 3′ ends of the 18S rRNA precursors terminating within the ITS sequence observed here for the *rps10* mutant (i.e. those giving the 450-bp band in cRT-PCR) showed a pattern similar to that previously reported for the mtPNPase-deficient mutant (PNP-) [48]. This resemblance led us to hypothesize that the aberrant processing of the 3′ end of 18S rRNA precursors in *rps10* mitochondria could be due to a compromised action of mtPNPase.

### Differential defects in 3′ end processing of mitochondrial rRNAs in *rps10*: severe for 18S, mild for 26S, almost absent for 5S

To test whether the reduced abundance of mitoribosomal protein uS10m in *rps10* specifically impairs 3′ end processing of 18S rRNA or also affects the maturation of the 3′end of other mitochondrial rRNAs, we compared the efficiency of 3′ end formation for mature 18S, 5S, and 26S rRNAs. Processing efficiency was estimated by calculating the ratio of mature rRNA to its precursor, both quantified by RT-qPCR within the same sample. A lower ratio indicates reduced processing efficiency. Previous studies have shown that mtPNPase contributes to the 3′ end formation of both 18S and 5S rRNAs [49], although its role in 26S rRNA maturation remains unclear. To address this, we included the Arabidopsis mutant line *amiR-PNP-3*, which exhibits reduced mtPNPase expression due to artificial microRNA-mediated silencing of the enzyme [29]. Notably, despite being homozygous, *amiR-PNP-3* plants show varying intensities of phenotypic alterations and considerable variability in mtPNPase transcript levels (see Supplementary Fig. S3 and Supplementary Fig. S8). Therefore, we selected plants that exhibited notable phenotypic alterations and at least 30% reduction in mtPNPase transcript levels when compared to WT for further analysis.

**Figure 3.**
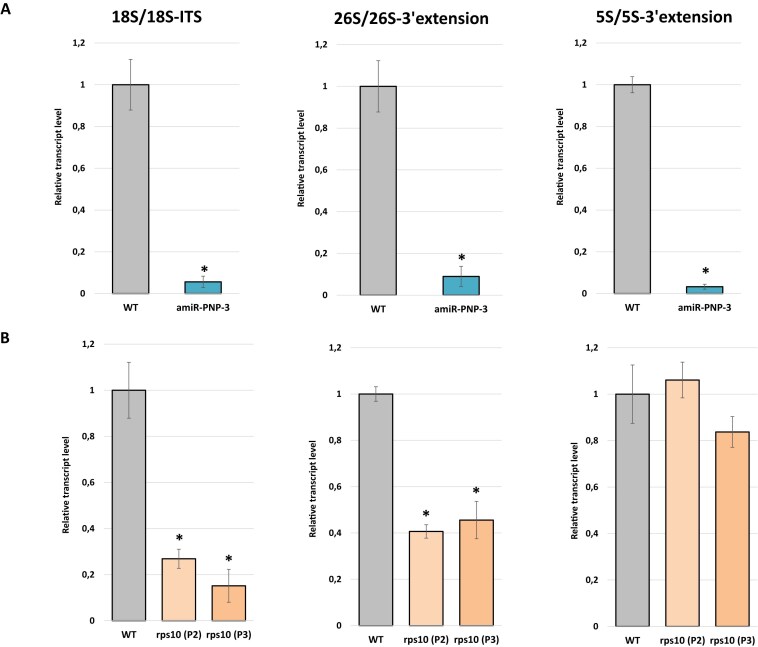
Efficiency of 3′ end processing of 18S, 26S, and 5S pre-rRNAs in *rps10* and *amiR-PNP-3* plants compared with WT**. (A**) *amiR-PNP-3*. (**B**) *rps10*. Efficiency is expressed as the ratio of mature transcript abundance to its precursor with a 3′ extension. Transcript levels were quantified by RT-qPCR and normalized to WT (set to 1). Data represent means of at least three biological replicates; error bars indicate standard deviation before normalization. Statistically significant differences from WT are marked with an asterisk (Student’s *t*-test, *P* <.05).

As shown in Fig. 3A, silencing of mtPNPase expression leads to a pronounced reduction in the 3′ end processing efficiency of all three examined mitochondrial rRNAs (mtrRNAs). These results clearly demonstrate the involvement of mtPNPase in the 3′-end formation of 18S, 26S, and 5S rRNAs. Next, we assessed the efficiency of 3′ end processing of mtrRNAs in the *rps10* mutant. The data indicate that 18S rRNA maturation is most severely affected, with only ∼20% of precursor transcripts undergoing correct 3′ end processing (Fig. 3B). In comparison, 26S rRNA displays a moderate defect (∼45% correctly processed), whereas 5S rRNA shows no statistically significant alteration in 3′ end processing in *rps10* mutants (Fig. 3B). This differential impact was highly reproducible and emphasizes a hierarchy of sensitivity among mitochondrial rRNAs to uS10m-dependent perturbations that inhibit mtPNPase functionality.

**Figure 4.**
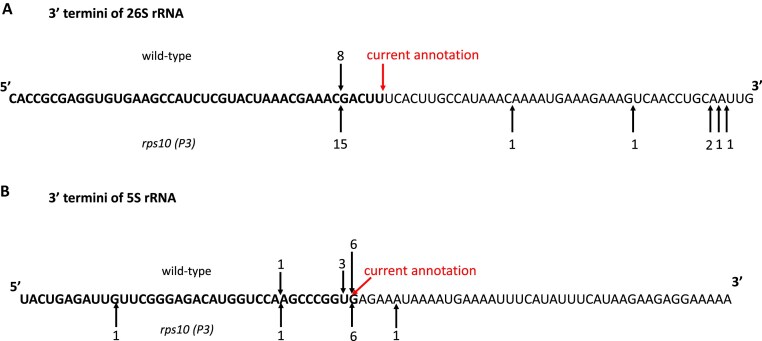
Mapping of 3′ termini of 26S rRNA and 5S rRNA by cRT-PCR in *rps10* (P3) and WT. (**A**) 3′termini of 26S rRNA**. (B**) 3′ termini of 5S rRNA. Black arrows indicate termini identified in individual clones, with clone numbers above. Red arrows mark annotated 3′ ends [47]; bold sequence denotes mature rRNA.

To further characterize rRNA processing defects, we mapped the 3′ ends of 26S and 5S rRNAs in WT and *rps10* plants using comparative cRT-PCR. Amplified products were cloned and sequenced to determine precise termini (Fig. 4, Supplementary Fig. S2, and Supplementary Table S3). The analysis revealed that the annotated 3′ end of 26S rRNA [47] is five nucleotides longer than the predominated terminus observed in both genotypes. In WT, all sequenced 26S rRNA molecules shared the same 3′ end, whereas in *rps10* (P3) ∼30% of sequenced clones exhibited extended 3′ ends, ranging from 20 to 45 nucleotides beyond the WT terminus. For 5S rRNA, the majority of clones in both WT and *rps10* (P3) mutants terminated at the annotated 3′ end [47], with ∼40% of sequenced clones being even slightly shorter in WT, whereas in *rps10* (P3) mutants only one out of nine transcripts exhibited a four-nucleotide 3′ end extension. Together with the quantitative data shown in Fig. 3B, these results reinforce the notion of differential sensitivity among mitochondrial rRNAs in *rps10* mutants.

### Multiple *in vivo* assays indicate that mtPNPase efficiency is decreased in *rps10* mitochondria

Our findings indicate that 3′ end processing of 18S and 26S rRNAs by mtPNPase is significantly impaired in *rps10* mitochondria, suggesting reduced enzymatic efficiency of mtPNPase in this mutant background. Although mtPNPase also contributes to the 3′ end maturation of 5S rRNA, as confirmed by our data (Fig. 3A), this process appears to be almost unaffected in *rps10*, for reasons that currently remain unclear. To further investigate whether the defect in 3′ end processing observed in *rps10* extends beyond rRNAs, we examined the behavior of other known mtPNPase substrates. This allowed us to assess whether the impaired processing reflects a broader disruption of mtPNPase-dependent RNA metabolism or is limited to specific transcript classes.

**Figure 5.**
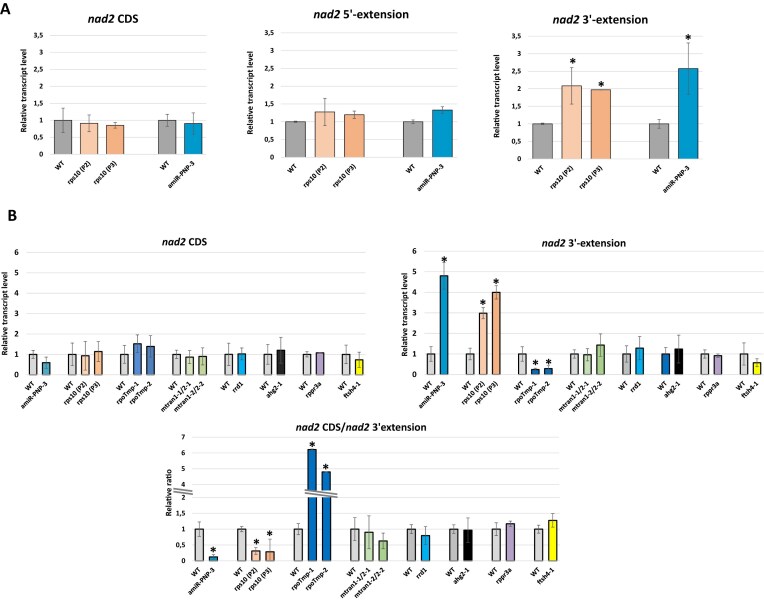
Accumulation and 3′-end processing efficiency of *nad2* transcripts in mitochondrial mutants. (**A**) RT-qPCR analysis of *nad2* transcripts in *rps10* and *amiR-PNP-3* plants. Primer pairs targeted the coding sequence (CDS), the region upstream of the mature 5′ end, and downstream of the mature 3′ end. (**B**) Levels of the mature *nad2*, its 3′ extended precursor, and processing efficiency (ratio of *nad2* to *nad2* 3′extension) in diverse mitochondrial mutants. For this panel, *amiR-PNP-3* and *rps10* mutants were reanalyzed, resulting in slight differences from panel (A). Transcript levels are normalized to WT (set to 1). Data represent means of at least three biological replicates; error bars indicate standard deviation before normalization. Statistically significant differences from WT are marked with an asterisk (Student’s *t*-test, *P *<.05). The *y*-axis break allows visualization of both low and high expression values.

We first quantified the abundance of 3′-extended *nad2* precursor RNA using RT-qPCR, an assay used previously to estimate the activity of mtPNPase in plants with a reduced level of this enzyme (*amiR-PNP-3*) [29]. In *rps10* mutants, the 3′-extended *nad2* transcript accumulated to higher levels in both *amiR-PNP-3* and *rps10* mutants, whereas the abundance of the mature *nad2* transcript remained largely unchanged relative to WT (Fig. 5A). The efficiency of 3′ end processing, expressed as the ratio of mature *nad2* to the 3′-extended *nad2* precursor is presented in Fig. 5B and indicates that in *rps10*, only ∼30% of *nad2* precursor molecules are properly processed at the 3′ end, further supporting the conclusion that mtPNPase activity is compromised in this mutant background. Furthermore, re-analysis of our earlier mitochondrial RNA sequencing (mtRNA-seq) data [2] supported the accumulation of reads downstream of the conventional 3′ end of the *nad2* transcript in *rps10* (Supplementary Fig. S4A).

**Figure 6.**
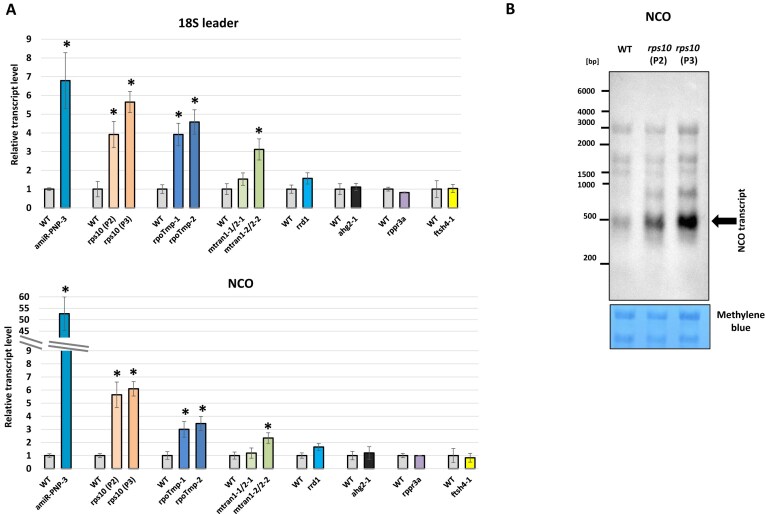
Accumulation of noncoding RNAs (18S rRNA leader, NCO) in mitochondrial mutants. (**A**) Accumulation of the 18S rRNA leader and NCO transcript. Transcript levels were quantified by RT-qPCR and normalized to WT (set to 1). Data represent means of at least three biological replicates; error bars indicate standard deviation before normalization. Statistically significant differences from WT are marked with an asterisk (Student’s *t*-test, *P* <.05). The *y*-axis break allows visualization of both low and high expression values. (**B**) Northern blot detection of the NCO transcript in WT and *rps10* using a 5′-biotinylated probe; methylene blue staining serves as loading control.

Beyond its role in mRNA processing, mtPNPase is also involved in RNA degradation. In Arabidopsis, mtPNPase is known to degrade the 18S rRNA leader sequence, which is a by-product of endonucleolytic cleavage upstream of the mature 18S rRNA [48], as well as the 500-nt NCO transcript [49]. To assess whether these degradation processes are also affected in *rps10*, we measured the abundance of both RNA species in *amiR-PNP-3* and *rps10* plants. As expected, RT-qPCR revealed a strong accumulation of both the 18S leader sequence and the NCO transcript in *amiR-PNP-3* (Fig. 6A). Notably, a significant increase was also observed in *rps10*. These findings are further supported by mtRNA-seq data (Supplementary Fig. S4B and C), which confirms the accumulation of both transcripts in *rps10*. In addition, Northern blot analysis verified that the NCO transcript was readily detectable in *rps10*, whereas it was barely detectable in WT mitochondria (Fig. 6B). Collectively, these results provide strong evidence that the degradation function of mtPNPase is impaired in *rps10* mitochondria, supporting the conclusion that uS10m deficiency negatively impacts mtPNPase activity in *vivo*.

### Accumulation of mtPNPase substrates in *mtran* and *rpoTmp* mutants

To find out if accumulation of mtPNPase substrates is unique to *rps10*, we determined it in several other mitochondrial mutants. We examined: (i) the *mtran1-1/2–1* and *mtran1-2/2–2* double mutants in mtSSU-specific ‘mitochondrial translation factors’ (mTRAN) PPR-like proteins, that, like uS10m, are required for mitochondrial translation [28]; (ii) the *rppr3a* mutant deficient in the rPPR3a mitoribosomal protein which is, however, functionally redundant with rPPR3b [19]; (iii) poly(A)-specific ribonuclease mutants (*rrd1* and *ahg2-1*) which accumulate polyadenylated mitochondrial RNAs [31, 32]. The latter were chosen to determine if an overabundance of polyadenylated transcripts, which are mtPNPase substrates, could be responsible for the decreased mtPNPase efficiency observed in *rps10*. Finally, we investigated (iv) *rpoTmp-1* and *rpoTmp-2* mutants deficient in dual-targeted mitochondrial and plastid RNA polymerase [25], as well as (v) the *ftsh4-1* mutant unrelated to RNA metabolism but lacking the inner-membrane mitochondrial protease FTSH4 [24]. For comparison and as a control, the *amiR-PNP-3* mutant with substantially reduced mtPNPase abundance was included.

**Figure 7.**
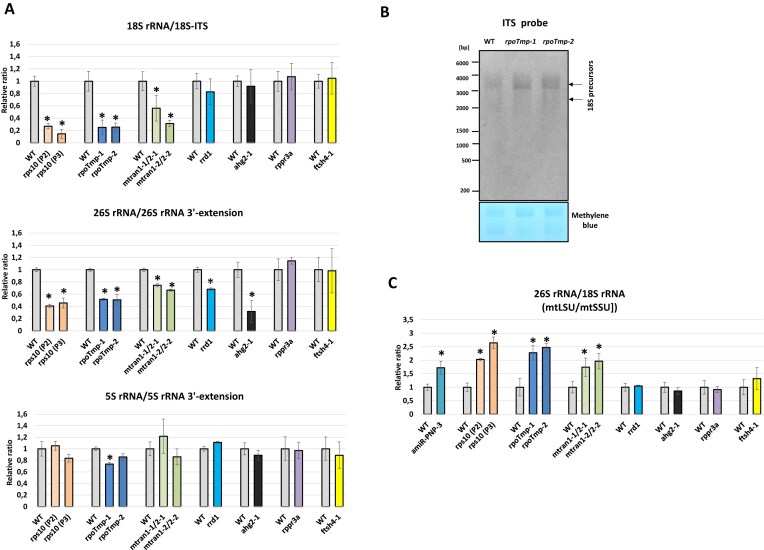
Relative efficiency of 3′ end processing of 18S, 26S, and 5S rRNA precursors and mtLSU/mtSSU ratio in mitochondrial mutants. (**A**) Processing efficiency (ratio of mature rRNA to its 3′ extended precursor) is expressed relative to that in the appropriate WT set to 1. Transcript level was determined by RT-qPCR. Data represent means of at least three biological replicates; error bars indicate standard deviation before normalization. Statistically significant differences from WT are marked with an asterisk (Student’s *t*-test, *P* <.05). (**B**) Northern blot detection of 18S rRNA precursors in WT, *rpoTmp-1*, and *rpoTmp-2*. A 5′-biotinylated probe complementary to the ITS region (gray dash in Fig. 2A) was hybridized to mitochondrial RNA. Arrows mark precursor forms; methylene blue staining serves as a loading control. (**C**) Ratio of mtLSU to mtSSU based on 26S and 18S rRNA abundance determined by RT-qPCR and expressed relative to WT (set to 1). Data represent means of ≥3 biological replicates; error bars indicate standard deviation before normalization. Statistically significant differences from WT are marked with an asterisk (Student’s *t*-test, *P* <.05).

Given the markedly reduced efficiency of 3′ end processing of 18S and 26S rRNA, but not 5S rRNA, in *rps10* (Figs 3 and 4), we examined whether similar defects occur in other mutants. To address this, RT-qPCR analysis was performed (Supplementary Fig. S5), and processing efficiency was expressed as the ratio of mature transcript to precursor (Fig. 7A). Similar to *rps10*, a significant reduction in 3′ end processing of 18S and 26S rRNA was detected in *rpoTmp-1, rpoTmp-2, mtran1-1/2–1*, and *mtran1-2/2–2*, whereas 5S rRNA remained largely unaffected. In line with this, accumulation of unprocessed 18S rRNA precursors in the *rpoTmp* mutants (Fig. 7B), similar to *rps10* (Fig. 2B), was confirmed by Northern hybridization. Notably, 18S rRNA consistently showed the most severe impairment, followed by a reproducible but milder defect in 26S rRNA (Fig. 7A). This gradient of susceptibility was attenuated in *mtran* mutants, which displayed the weakest reduction in processing efficiency. Taken together, these observations reveal a hierarchy of sensitivity among rRNA species, suggesting that impaired 18S rRNA processing may represent the primary defect, with secondary effects on 26S rRNA and minimal influence on 5S rRNA. These findings point to a preferential dependence of 18S rRNA 3′-end processing on mtPNPase-mediated exonucleolytic trimming. Unexpectedly, two mutants accumulating polyadenylated mitochondrial transcripts, *rrd1* and *ahg2-1*, displayed a distinct pattern. A statistically significant reduction in 26S rRNA 3′ end processing was detected, while other rRNAs were unaffected (Fig. 7A). This impairment is particularly strong in *ahg2-1*.

RT-qPCR was also employed to quantify the accumulation of mtPNPase degradation substrates across mutants. A statistically significant accumulation of two noncoding transcripts, the 18S rRNA leader and the NCO transcript, was detected in *amiR-PNP-3, rps10, mtran1-2/2–2*, and both *rpoTmp* mutants, but not in *mtran1-1/2–1*, which retains detectable mTRAN1 protein and exhibits milder phenotypic defects compared to *mtran1-2/2–2* [28] (Fig. 6A). In addition, the 3′-extended *nad2* mRNA was analyzed for both steady-state abundance and processing efficiency. Markedly reduced 3′ end maturation was observed exclusively in *amiR-PNP-3* and *rps10* mutants (Fig. 5), indicating that only in these mutants the 3′ end processing of *nad2* mRNA is impaired. In contrast, in *rpoTmp* mutants, *nad2* shows a higher mature-to-precursor ratio, with increased mature RNAs and reduced precursors. As RPOTmp is likely the polymerase responsible for *nad2* transcription in Arabidopsis mitochondria [25], its absence suggests that *nad2* transcription in *rpoTmp* is carried out by RPOTm. This polymerase shift results in a markedly elevated CDS/3′-extension ratio, suggesting that RPOTm couples more effectively with the *nad2* 3′-processing machinery.

### Increased 26S/18S rRNA ratio in mutants with a reduced mtPNPase efficiency

Among the features of the mitoribosome population in *rps10*, a pronounced excess of 26S relative to 18S rRNA was observed, a pattern compatible with an imbalance in mitoribosomal subunits [3]. As revealed by RT-qPCR, a significantly increased ratio of 26S rRNA to 18S rRNA was also found in *amiR-PNP-3* and other mutants with the reduced mtPNPase efficiency (*rpoTmp-1, rpoTmp-2, mtran1-1/2–1, mtran1-2/2–2* (Fig. 7C).

In *amiR-PNP-3*, 18S rRNA level decreased to ∼50% of WT, whereas 26S rRNA abundance did not differ significantly from WT (Supplementary Fig. S5) resulting in a 26S/18S ratio of ∼1.7 (Fig. 7C). The pronounced reduction in 18S rRNA abundance in *amiR-PNP-3* argues against a role for mtPNPase in the degradation of mature 18S rRNA, as such a function would be expected to cause its accumulation. Instead, this pattern—reduced levels of mature 18S rRNA accompanied by a marked accumulation of the 18S–ITS precursor—is fully consistent with defective 18S rRNA maturation caused by reduced mtPNPase activity. In *rps10* and *rpoTmp* mutants both rRNAs were more abundant than in WT, but the increase was greater for 26S rRNA (Supplementary Fig. S5), resulting in a 26S/18S ratio of ∼2.5 (Fig. 7C). In *mtran1-1/2–1* and *mtran1-2/2–2*, the increase in 26S rRNA was slightly greater than the decrease in 18S rRNA (Supplementary Fig. S5), yielding a ratio of ∼2.0 (Fig. 7C).

As the RT-qPCR primers used for 18S and 26 rRNAs amplify not only mature rRNAs but also precursor forms, the absolute values 26S/18S ratios should be interpreted with caution. However, in *rps10, rpoTmp*, and *mtran* comparable accumulation of pre-18S rRNA and pre-26S rRNA is observed (∼10-fold, 6-fold, and 2-fold, respectively) (Supplementary Fig. S5), indicating that precursor co-amplification is unlikely to distort the 26S/18S ratio in these backgrounds. Moreover, precursors represent only a minor fraction relative to mature rRNAs, making their contribution to the ratio negligible. A specific situation was observed in *amiR-PNP-3*. Pre-18S rRNA accumulates much more strongly than pre-26S rRNA, meaning that the measured 26S/18S ratio (∼1.7) may underestimate the true imbalance. Despite these methodological considerations, all examined mutants display an increased 26S/18S ratio, pointing to a consistent alteration in mitochondrial rRNA processing when mtPNPase function is compromised.

### mtPNPase expression is increased in mutants with a reduced mtPNPase efficiency

**Figure 8.**
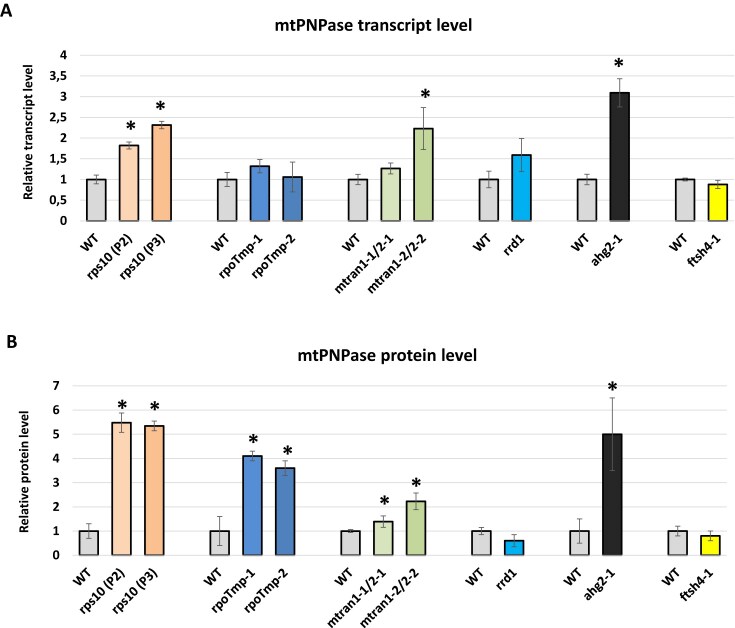
mtPNPase expression in mitochondrial mutants. (**A**) Relative transcript levels determined by RT-qPCR in total RNA from several mutants; values normalized to WT (WT = 1). (**B**) mtPNPase protein abundance assessed by immunoblotting of equal amounts of mitochondrial protein using anti-mtPNPase antibodies; representative blots are shown in Supplementary Fig. S6. Bands intensities were quantified with ImageJ. Data represent means of at least three biological replicates; error bars indicate standard deviation before normalization. Statistically significant differences from WT are marked with an asterisk (Student’s *t*-test, *P* <.05).

Looking for the cause of the decreased *in vivo* efficiency of mtPNPase, we determined its expression at the transcript and protein levels. Contrary to expectation, we found an increased abundance of both the transcript and protein in *rps10* and *mtran1-2/2–2* and only at the protein level in both *rpoTmp* mutants; in *mtran1-1/2–1* the protein and transcript level was increased slightly (Fig. 8 and Supplementary Fig. S6). In all the other mutants, with the exception of *ahg2-1*, the mtPNPase expression was similar to that in the WT. Thus, the reduced mtPNPase efficiency observed in *rps10, mtran1-2/2–2*, and the two *rpoTmp* mutants is not caused by a reduced expression of the enzyme. On the contrary, one could claim that a compensatory mechanism is in action, increasing mtPNPase abundance in response to its reduced effectiveness.

### Changes in mtPNPase and rRNA distribution in *rps10* between the soluble and membrane fractions

**Figure 9.**
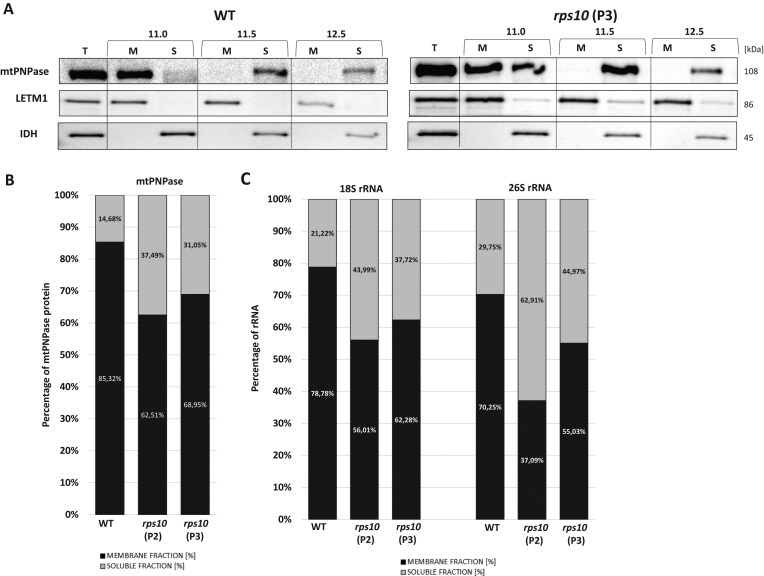
Distribution of mtPNPase and rRNAs in membrane and soluble fractions of mitochondria in *rps10* compared with WT. (**A**) Mitochondria from WT and *rps10* were extracted with sodium carbonate at pH 11.0, 11.5, or 12.5, and 50 µg of protein was separated into membrane (M) and soluble (S) fractions by centrifugation. Fractions were analyzed by SDS–PAGE and immunoblotting with antibodies against the indicated proteins. LETM1 is an integral inner membrane marker; IDH is a soluble matrix marker. T, total load of 50 µg untreated mitochondria. MW markers are shown in kDa. (**B**) Quantification of mtPNPase in M and S fractions. Band intensities were measured with ImageJ and averaged from four independent experiments; values are expressed as the percentage of total protein. (**C**) Distribution of 18S and 26S rRNAs in M and S fractions of mitochondria separated by freeze–thawing.18S and 26S rRNAs were quantified by RT-qPCR and averaged from ≥3 independent experiments; values are expressed as percentage of total rRNA.

The association of the *E. coli* RNA degradosome with the inner cytoplasmic membrane bears a substantial effect on its functioning [50, 51]. These findings prompted us to compare the distribution of Arabidopsis mtPNPase in the soluble and membrane mitochondrial fractions between the WT and the *rps10* mutant. To this end we used carbonate extraction, a method widely used to separate integral membrane proteins from peripheral membrane and soluble proteins [52]. The LETM1 (leucine zipper-EF-hand-containing transmembrane protein 1) was used as a marker of the membrane fraction (M), and IDH as a marker of the soluble fraction (S). Alkaline extraction at pH 11.0 showed that mtPNPase was present in both the membrane and the soluble fraction in both the WT and *rps10*. However, its amount in the soluble fraction was substantially higher in *rps10* (Fig. 9A and B). In contrast to *rps10*, in the remaining mutants showing a defect in 18S pre-rRNA processing (*mtran 1–1/2–1; mtran1-2/2–2, rpoTmp-1*, and *rpoTmp-2*) mtPNPase was found, similarly to the WT, mainly in the membrane fraction (Supplementary Fig. S7). Upon extraction at a higher pH (11.5 or 12.5) mtPNPase was recovered only in the soluble fraction in both the WT and *rps10* (Fig. 9A). These data demonstrate that while most mtPNPase is tightly associated with the membrane, it is not an integral membrane protein, regardless of genotype.

Rapid freezing and thawing of mitochondria followed by ultracentrifugation is another method for separating membrane proteins from soluble ones [44]. Using this method, the amount of 18S rRNA and 26S rRNA (as a proxy for the respective mitoribosome subunits, see above) in the membrane and soluble fraction was determined by RT-qPCR. Only 20%–30% of the mitochondrial rRNAs were found in the soluble fraction in WT mitochondria, as shown in Fig. 9C, and roughly twice as much in *rps10*. In conclusion, a substantially larger fraction of both mtPNPase and mitoribosomal subunits is not associated with the membrane in *rps10* when compared to WT mitochondria.

### Macroscopic phenotype of *amiR-PNP-3* is similar to mutants accumulating mtPNPase substrates (*rps10, mtran, rpoTmp*)

Under long-day conditions, the *amiR-PNP-3* mutant exhibited delayed growth and development, with wrinkled or curled rosette leaves, consistent with previous observations by [29]. We also found phenotypic differences between *amiR-PNP-3* and the WT; however, phenotypic variability within *amiR-PNP-3* populations complicates the description of a uniform phenotype: some plants were markedly smaller and developmentally delayed (Supplementary Fig. S8A, upper panel), whereas others were similar in size to WT (Supplementary Fig. S8A, bottom panel), with the proportion of these phenotypic classes varying among populations. The mutants showed wrinkled or curled leaves late in vegetative growth (Supplementary Fig. S8A), and some plants displayed shorter inflorescences.

Similar to *amiR-PNP-3*, Arabidopsis mutants that accumulate mtPNPase substrates (*rps10, mtran 1–1/2–1, mtran1-2/2–2, rpoTmp-1*, and *rpoTmp-2*) exhibit reduced size and delayed development, consistent with earlier reports [23, 25, 28] (Supplementary Fig. S8B). Notably, *rps10* and *rpoTmp* mutants, which accumulate mtPNPase substrates more extensively than *mtran*, frequently display wrinkled or curled rosette leaves, a feature also observed in *amiR-PNP-3* (Supplementary Fig. S8B). The rosette leaf morphology observed in *rps10, rpoTmp*, and *amiR-PNP-3* resembles the leaf characteristics described for mutants with impaired mitochondrial function [25, 53]. Reduced mtPNPase activity alone can induce such morphological changes, but in *rps10* or *rpoTmp* the altered leaf phenotype likely reflects a combined effect of mtPNPase deficiency and gene-specific mitochondrial dysfunction.

## Discussion

**Figure 10.**
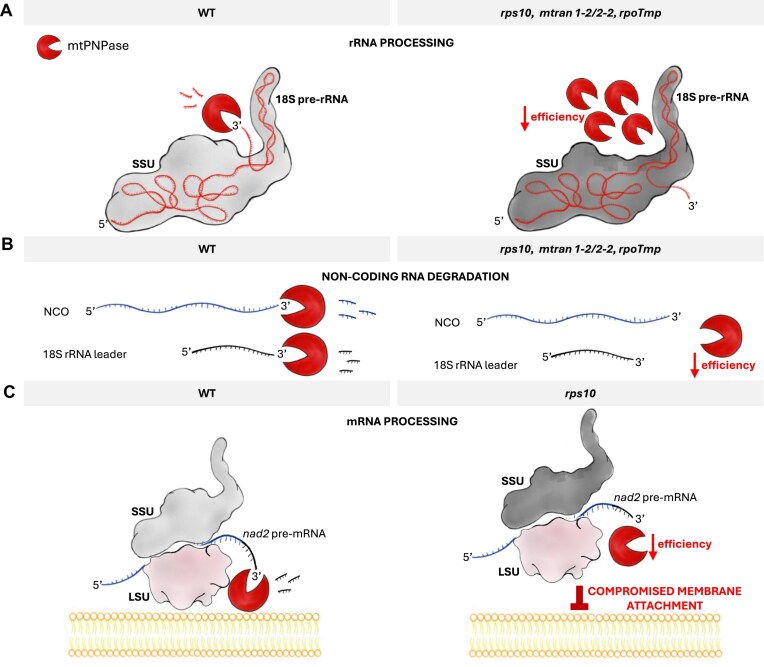
Model of impaired RNA processing and degradation caused by reduced mtPNPase efficiency in Arabidopsis mutants defective in 18S pre-rRNA maturation. (**A**) Processing of the 3′ end of 18S pre-rRNA in WT and mutants (*rps10, mtran1-2/2–2, rpoTmp*). Misfolded mtSSUs alter18S pre-rRNA folding, limiting mtPNPase access to its 3′ end and sequestering the enzyme. (**B**) Degradation of noncoding RNAs (NCO and 18S rRNA leader) in WT and mutants (*rps10, mtran1-2/2–2, rpoTmp*). In mutants, degradation is inefficient because mtPNPase is trapped in reaction (**A**). (**C**) Processing of 3′-extended *nad2* pre-mRNA in WT and *rps10*. Reduced processing in *rps10* results from weaker mtPNPase association with the membrane due to altered mitoribosome structure. Misfolded mtSSUs are shown in dark gray; WT mtSSUs in light gray; 18S pre-rRNA in red.

This study demonstrates that disruption of mitochondrial small subunit biogenesis in Arabidopsis, associated with impaired maturation of the 3′ end of 18S pre-rRNA in *rps10, rpoTmp*, and *mtran* mutants, results in the accumulation of several RNAs normally processed or degraded by mtPNPase, a key enzyme of mitochondrial RNA metabolism. The reduction in mtPNPase efficiency observed in these mutants *in vivo* is not explained by decreased expression or catalytic impairment but instead we propose that it arises from sequestration of mtPNPase at sites of 18S rRNA 3′-end processing, restricting the enzyme’s access to other substrates and thereby slowing their breakdown (Fig. 10A and B). Although mtPNPase protein levels rise markedly in these mutants, this compensation is ineffective because persistent misprocessing of 18S pre-rRNA compromises overall enzymatic efficiency. These findings reveal a functional link between rRNA maturation and RNA decay, highlighting the importance of ribosome biogenesis for transcriptome homeostasis in plant mitochondria.

**Figure 11.**
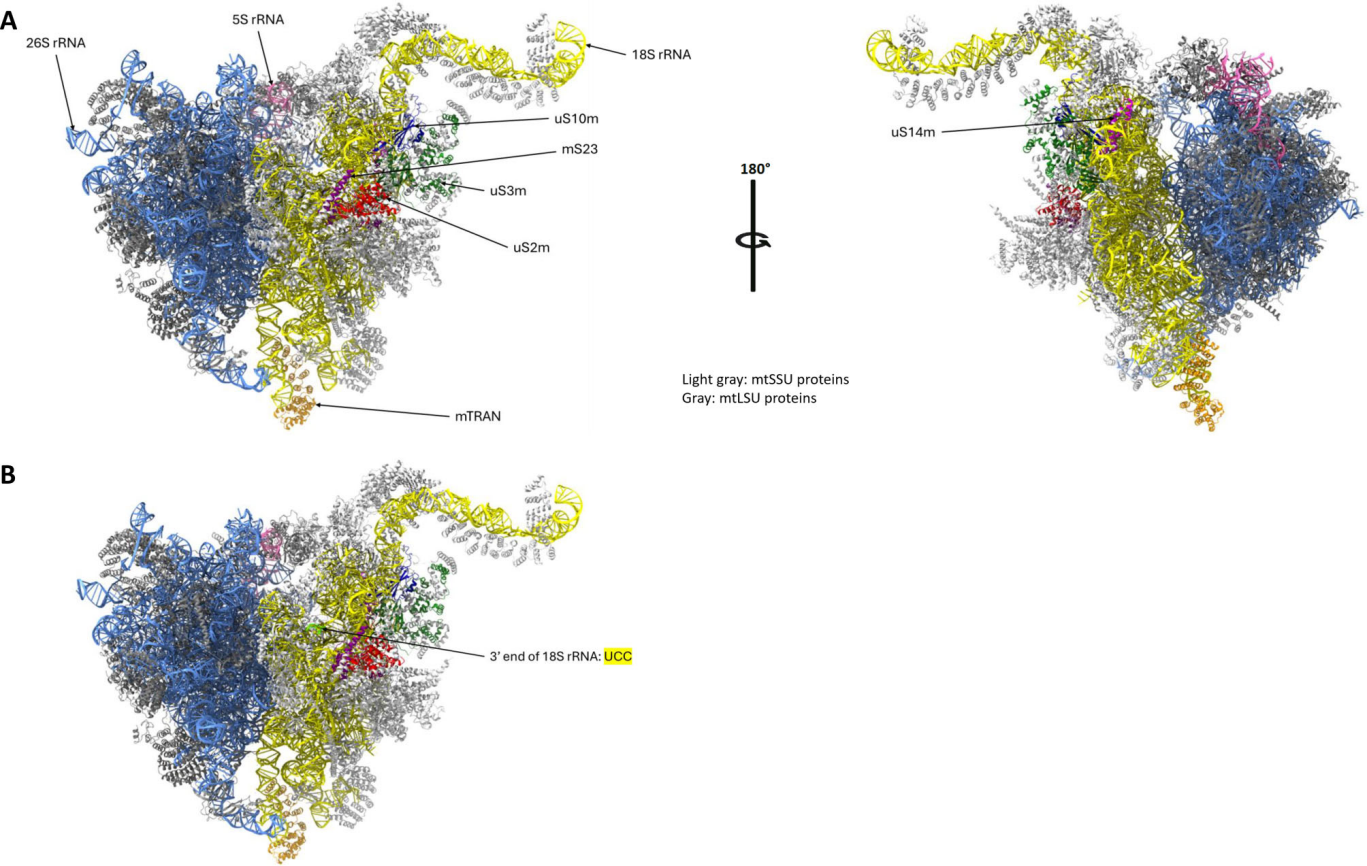
Localization of uS2m, uS3m, mS23, uS14m, uS10m and mTRAN within the mitochondrial small subunit (mtSSU). The overall model of plant mitoribosome [55] with highlighted (**A**) individual proteins (uS2m, uS3m, ms23, uS14m, uS10m, and mTRAN) and (**B**) 3′ end of the 18S rRNA. 26S, 18S and 5S rRNAs are shown in cornflower blue, yellow and magenta, respectively. Other ribosomal proteins are shown in gray (large subunit, LSU) and light gray (small subunit, SSU).

Our results indicate that in the *rps10* mutant, the defective processing of 18S pre-rRNA concerns only the 3′ terminus, which is processed by mtPNPase, while the correct 5′ end is produced by a currently unidentified endonuclease. The *rps10* mutation reduces the abundance not only of the uS10m protein, but also that of six additional mtSSU proteins by an indirect mechanism. According to the structural model of mtSSU [54], the proteins present at a substoichiometric level in *rps10* (uS2m, uS3m, mS23, uS14m, and uS10m) cluster around the 3′ end of the 18S rRNA, which fails to undergo proper processing in the mutant (Fig. 11). The subunit localization of the remaining two depleted proteins, mS84 and mS85, has not been established. mTRAN is placed at the foot of mtSSU (Fig. 11), which is not near the 3′ end of 18S rRNA but near the 5′ end, and the authors propose that mTRAN stabilizes the 18S rRNA [55]. Even when positioned at the 5′ end, loss of mTRAN may induce long-range perturbations across the rRNA scaffold, that alter the conformation of the head domain where the 3′ terminus resides. The relatively weak effect on 18S rRNA processing compared with the deficiency of S10 is consistent with long-range mechanism.

We found that a deficit in mtSSU mitoribosomal proteins *(rps10, mtran*) or a loss of the RPOTmp polymerase (*rpoTmp*) leads to the accumulation of incompletely processed 18S rRNA precursors. In human ribosomes, 18S rRNA assembles with 33 ribosomal proteins to form the SSU, and deficiency in any of 31 of those proteins leads to the accumulation of 18S rRNA precursors [56]. Therefore, it is not unexpected that a depletion of uS10m (*rps10*) or mTRAN1/mTRAN2 (*mtran1-1/2–1; mtran1-2/2–2*) results in the accumulation of immature 18S rRNA transcripts in plant mitochondria. By contrast, the molecular basis for the 18S rRNA 3′-end processing defect observed in *rpoTmp* mutants is less clear. A possible insight comes from studies of eukaryotic nuclear RNA polymerase I, where reduced Pol I transcription rate induces defects in pre-rRNA processing and ribosome biogenesis [57]. By analogy, we hypothesize that when only one of the two mitochondrial RNA polymerases (specifically RPOTm, in the absence of RPOTmp) is functional, the rate of pre-rRNA elongation may decrease. This reduced transcription rate, in turn, impairs pre-rRNA processing. It should be emphasized, however, that this scenario is speculative and requires experimental validation, and that alternative explanations cannot be excluded. Furthermore, we propose that structural perturbations of 18S pre-rRNA caused by mitoribosomal protein depletion in *rps10* and *mtran*, or by hypothetically altered rDNA transcription rates in *rpoTmp* may hinder mtPNPase access to the 3′ end of rRNA, thereby compromising its exonucleolytic trimming.

An unexpected result of this study is the observation that *rps10, rpotmp*, and *mtran* mutants exhibit reduced mtPNPase activity not only toward the 18S pre-rRNA but also toward other substrates of this enzyme, including the 3′-end extension of the 26S rRNA and noncoding RNAs. Interestingly, pre-RNA processing defects were not uniform: 18S rRNA consistently exhibited the most severe impairment, 26S showed a moderate defect, and 5S remained nearly unaffected despite its high abundance. Although the magnitude of the effect varies between mutants, with *mtran1/2* showing milder changes, the hierarchy of rRNA processing sensitivity is preserved, suggesting that defects in mtSSU biogenesis preferentially compromise 18S rRNA processing, with lesser effects on 26S rRNA and minimal impact on 5S rRNA.

Like its human counterpart, plant mtPNPase is thought to localize to discrete foci [58]. We propose that mtPNPase becomes trapped at the sites of 18S pre-rRNA processing, where the incompletely processed 18S precursors act as molecular sinks, limiting the enzyme’s availability for other substrates. The selective inhibition of the 3′-end processing of the 26S rRNA contrasts with the unaffected maturation of the 5S rRNA and most likely reflects spatial compartmentalization of RNA metabolism. Because the 5S rRNA is co-transcribed with the 18S rRNA, it is likely processed within SSU-foci where mtPNPase is already enriched and remains accessible despite sequestration by incompletely processed 18S rRNA intermediates. In contrast, the 3′-end trimming of the 26S rRNA would require sufficient mtPNPase at LSU-foci, which may become depleted when the enzyme is sequestered at defective SSU processing sites.

The efficient maturation of the 5S rRNA 3′ terminus by mtPNPase contrasts with its impaired processing of the 18S rRNA 3′ end in *rps10, rpoTmp*, and *mtran* mutants, indicating that the observed defects in RNA processing and turnover are unlikely to arise from a loss of mtPNPase catalytic activity *per se*. A definitive exclusion of catalytic impairment will require *in vitro* assays using purified enzyme and defined labeled substrates. Our initial attempts to use whole mitochondrial extracts for this purpose yielded inconclusive results: although substrate degradation was reproducible, it lacked mtPNPase specificity, as substantial degradation was also detected in extracts deficient in mtPNPase. This suggests extensive interference from other ribonucleases.

In conclusion, we propose that the accumulation of mtPNPase substrates observed in *rps10, rpoTmp*, and *mtran* mutants is caused by a mechanism of spatial sequestration wherein mtPNPase becomes physically trapped by incompletely processed 18S rRNA intermediates at SSU-foci (Fig. 10A). The local enrichment of the enzyme leads to its reduced abundance at other foci (Fig. 10B). Consequently, while the total amount of mtPNPase increases, its effective availability for other substrates is severely diminished causing subsequent accumulation of mtPNPase-mediated substrates. This hypothesis provides a coherent explanation for our observations, but a direct *in vivo* demonstration of mtPNPase spatial sequestration is still lacking.

The spatial sequestration model mirrors the logic of the substrate overload model [59] but differs in its triggering mechanism. In human cells lacking the REXO2 oligoribonuclease, short mitochondrial RNAs accumulate [59]. Their excess occupies mtPNPase and in consequence reduces the pool of enzyme for global mitochondrial RNA degradation. In contrast, our data collectively suggest that the reduced mtPNPase efficiency in *rps10, rpoTmp*, and *mtran* may not simply reflect competitive substrate overload. Rather, the enzyme appears sequestered at the 18S rRNA 3′ processing site, limiting its availability for other mitochondrial substrates. Consistent with this, the elevated polyadenylated mitochondrial mRNA levels in *rrd1* and *ahg2-1* do not impair overall mtPNPase activity. These mutants show no significant accumulation of mtPNPase substrates, except for the 3′ extension of 26S rRNA (Supplementary Fig. S5). Thus, mtPNPase remains globally functional despite excess polyadenylated transcripts, contradicting the substrate-overload model. On the other hand, our results suggest that RRD1 [31] and AHG2 [32] may specifically cooperate with mtPNPase during 26S rRNA maturation (Fig. 7A).

Beyond the shared RNA-metabolism defects of *rps10, rpoTmp*, and *mtran* mutants, only *rps10* exhibits a shift of both mtPNPase and mitoribosomes from the membrane to the soluble fraction. Although the functional impact is unclear, parallels with bacterial degradosomes suggest a *rps10*-specific mode of mtPNPase regulation. In bacteria, degradosomes containing PNPase are associated with ribosomes, and membrane attachment is necessary for the enzyme efficiency *in vivo*, but not for its catalytic activity *in vitro* [51]. In plants, at least some mitoribosomes are tightly bound to the mitochondrial membrane [22], and mtPNPase associates with the mitoribosome, albeit not as an integral part of it [4, 5]. We postulate that, similarly to the degradosome in bacteria [60–62], the efficiency of plant mtPNPase *in vivo* depends on the membrane association modulated by interactions with mitoribosomes. We propose that weakened membrane association, likely a consequence of altered mitoribosome architecture, reduces mtPNPase efficiency in *rps10* (Fig. 10C). In different organisms, mitoribosomes attach to the membrane through various mechanisms, but always via the LSU. Our proteomics data indicate that not only the mtSSU assembly, but also that of mtLSU is disrupted in *rps10*, although to a much lesser extent. Because mtPNPase lacks a membrane-anchoring domain, translationally active mitoribosomes may serve as hubs for recruiting mtPNPase to process 3′-extended precursors of translated mRNAs, such as *nad2*. In agreement with this interpretation, the notable decrease in mtPNPase membrane association observed exclusively in *rps10* may explain why the accumulation of unprocessed *nad2* transcripts was detected only in this mutant and not in the others.

Mutants affecting mtSSU assembly share multiple molecular and also phenotypic features with mtPNPase-deficient line. All lines exhibit growth retardation, while *rps10* and *rpoTmp* mutants, those with higher substrate accumulation, often develop wrinkled or curled rosette leaves, a trait also seen in *amiR-PNP-3* (Supplemetary Fig. S8). The phenotype of *amiR-PNP-3* reflects severely reduced mtPNPase levels, whereas the phenotypes of *rps10, rpoTmp*, and *mtran* mutants likely result from mild mtPNPase deficiency combined with gene-specific mitochondrial dysfunction. In this context, it should be mentioned that embryo lethality of mtPNPase null mutants [10] and early silencing of homozygous *rps10* plants causing severe phenotypic abnormalities and premature death [23] underscore the essential roles of mtPNPase and uS10m in mitochondrial physiology. Together, these findings indicate that reduced mtPNPase efficiency, whether primary or secondary to mitoribosome defects, has clear physiological consequences.

Our findings suggest that accurate 18S pre-rRNA processing may be critical for sustaining mtPNPase functionality. We speculate that environmental stress, known to impair cytoplasmic rRNA maturation [63], could similarly affect mitochondrial rRNA processing, transiently limiting mtPNPase efficiency and reshaping the transcriptome. Such remodeling might provide adaptive benefits under conditions of compromised mitoribosome biogenesis.

## Supplementary Material

gkag609_Supplemental_Files

## Data Availability

The mass spectrometry proteomic data, including acquired raw files as well as output files from the MaxQuant search are deposited with the ProteomeXchange Consortium via the MassIVE partner depository (https://massive.ucsd.edu/) with the dataset identifier MSV000095401.
