# Supplementary material for: Dysregulated 3′-end processing of 18S pre-rRNA decreases mtPNPase efficiency in plant mitochondria
Source: Nucleic Acids Res. 2026 Jun 17;54(11):gkag609. doi: 10.1093/nar/gkag609 (PMC13273313; doi:10.1093/nar/gkag609)
Supplement: gkag609_Supplemental_Files [file gkag609_supplemental_files.zip › Supplementary Figures.pdf]

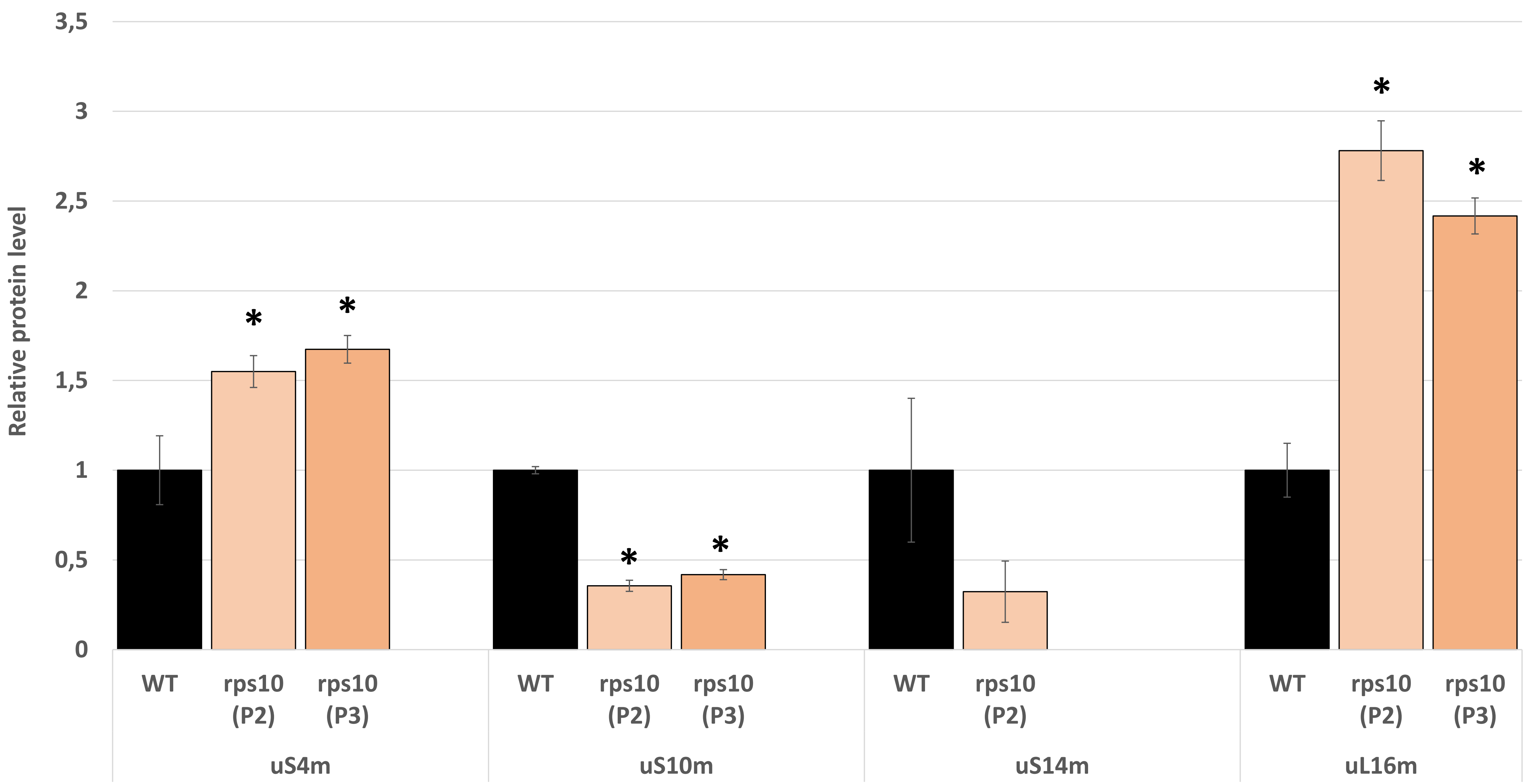

Supplementary Figure S1. Quantification of ribosomal proteins in mitochondrial fractions of *rps10* compared with wild-type plants. Band intensities were measured with ImageJ. Values are normalized to wild-type (WT=1). Data represent means of at least three biological replicates; error bars indicate standard deviation before normalization. Statistically significant differences from the WT are indicated by asterisk (Student's t test; $p < 0.05$). Quantitative data for uS14m are shown only for P2.

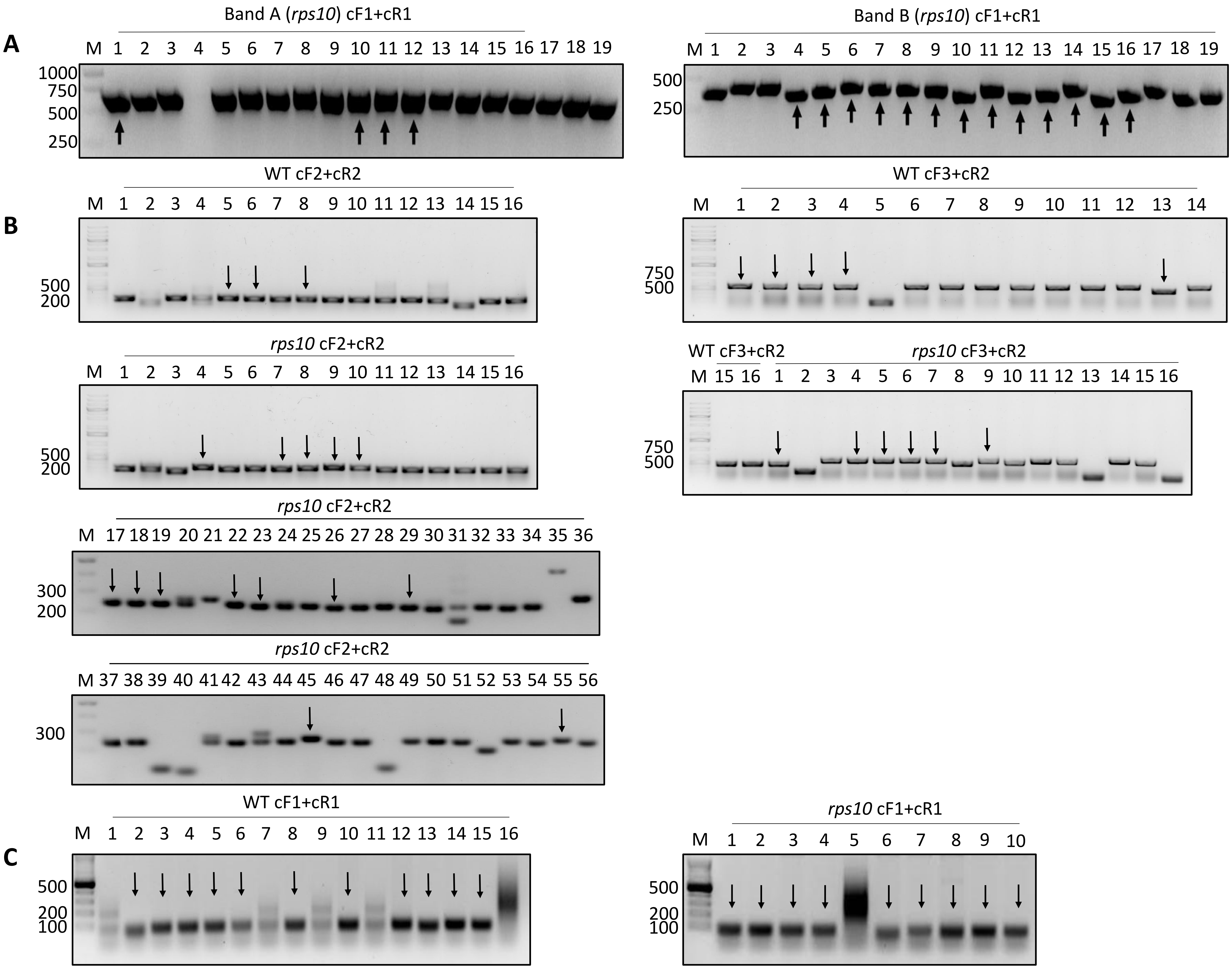

Supplementary Figure S2. Circular RT–PCR amplification products spanning junctions between the 5′ and 3′ ends of (A) 18S rRNA, (B) 26S rRNA, (C) 5S rRNA in *rps10* and wild-type. Agarose gels show fragments amplified with primer pairs specific for circularized RNA ends (primer sequences in Supplementary Table S1). Primer combinations are indicated above each gel. Two cRT–PCR reactions with different primers were used to investigate 3′ termini of 26S rRNA. For 18S rRNA, PCR with primers cF1 and cR1 yielded two bands (A and B; see Figure 2C); products from each band were sequenced. Size markers (bp) are shown on the left; arrows indicate sequenced products.

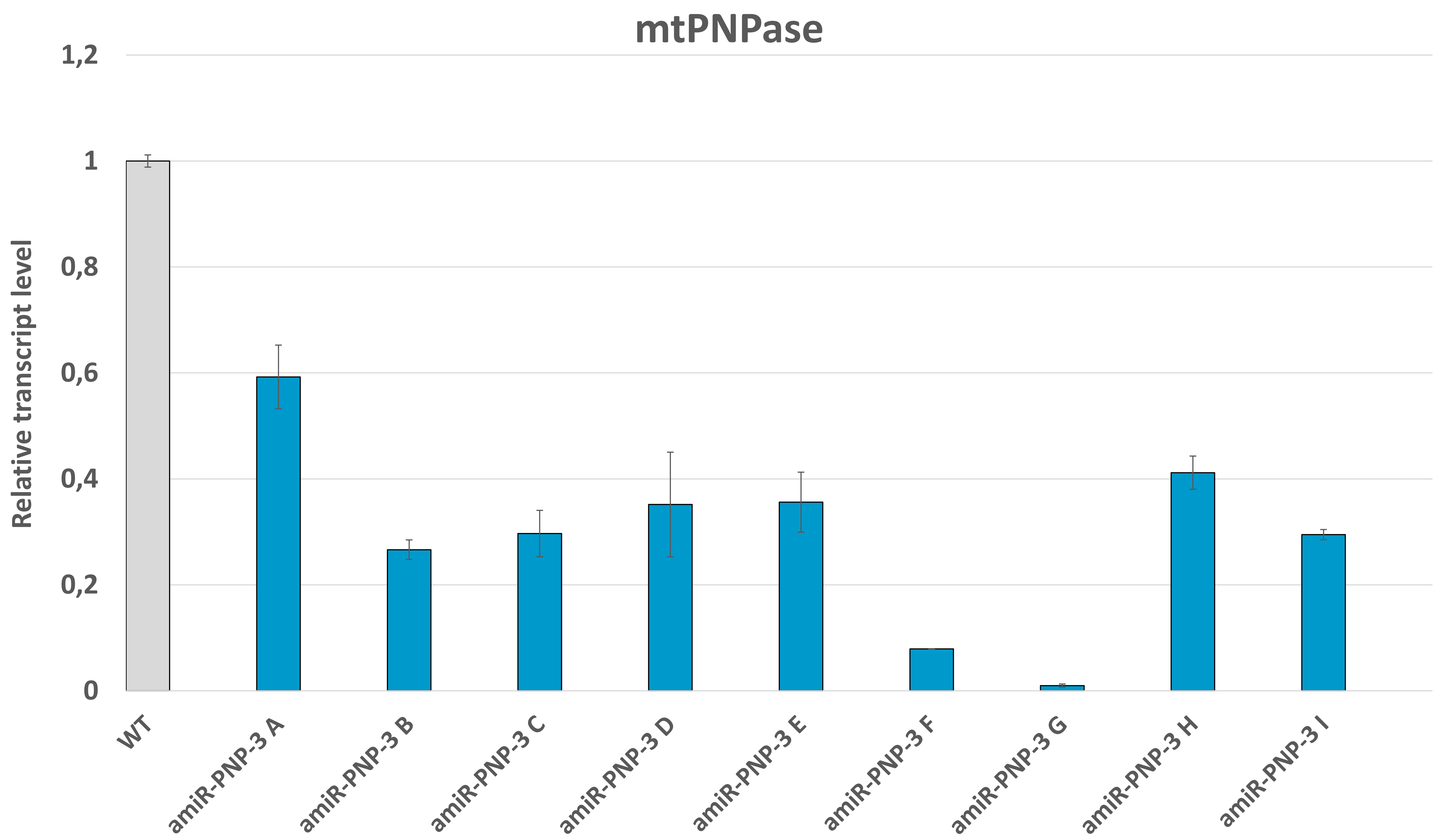

Supplementary Figure S3. RT-qPCR analysis of mtPNPase transcript levels in individual *amiR-PNP-3* plants. Transcript levels are shown relative to wild-type (set to 1). Data represent means of at least three biological replicates; error bars indicate standard deviation before normalization. Individual plants are identified by letters (A, B, C, etc.) added after the mutant name.

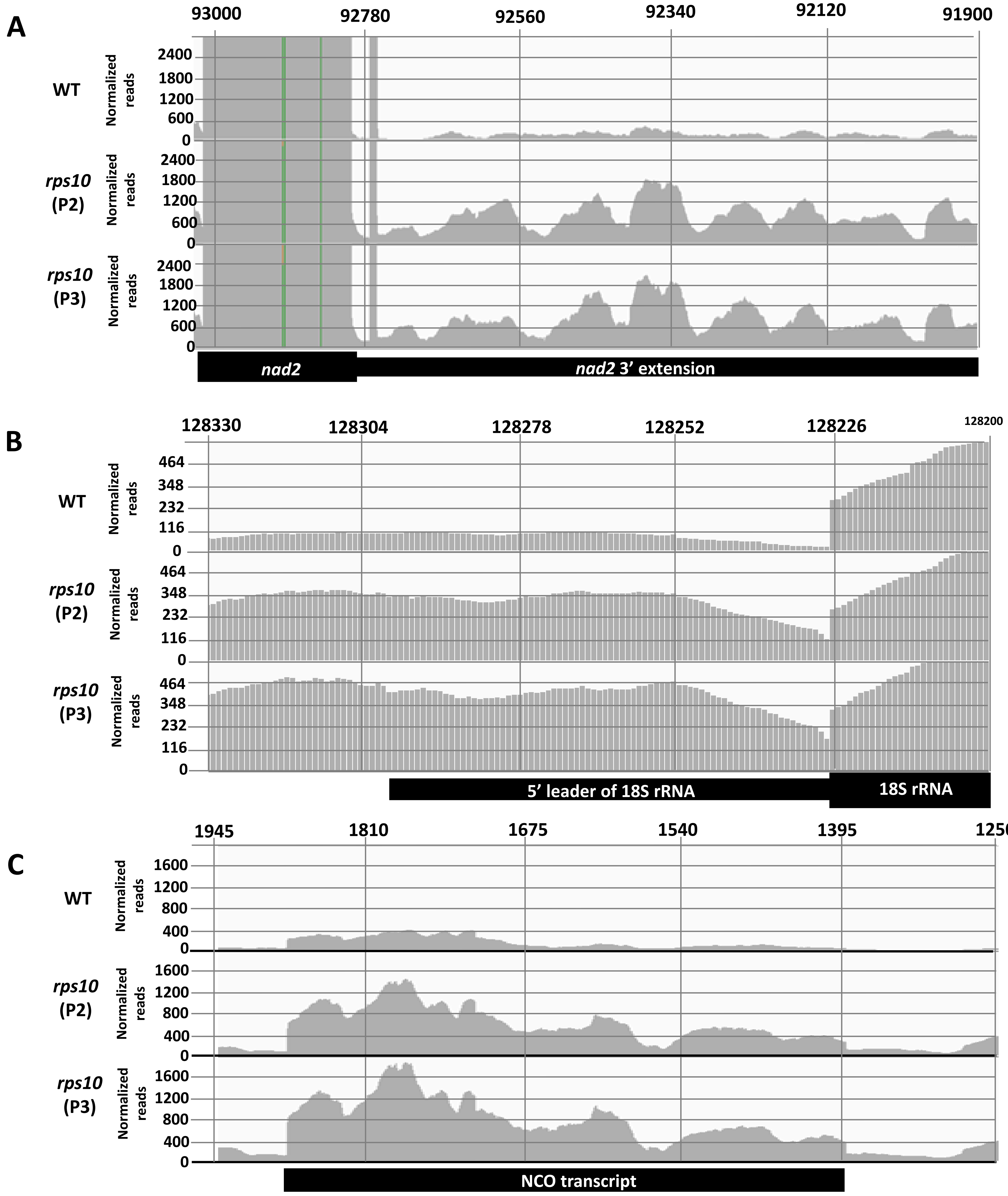

Supplemental Figure S4. Coverage of mtRNA-seq reads representing (A) 3'-extended *nad2*, (B) 5'leader of 18S rRNA, and (C) NCO transcript in *rps10* and wild-type. Reads mapping to the indicated regions were normalized to the total number of reads in mtRNA-seq libraries, and their distribution was visualized using Integrative Genomics Viewer (IGV).

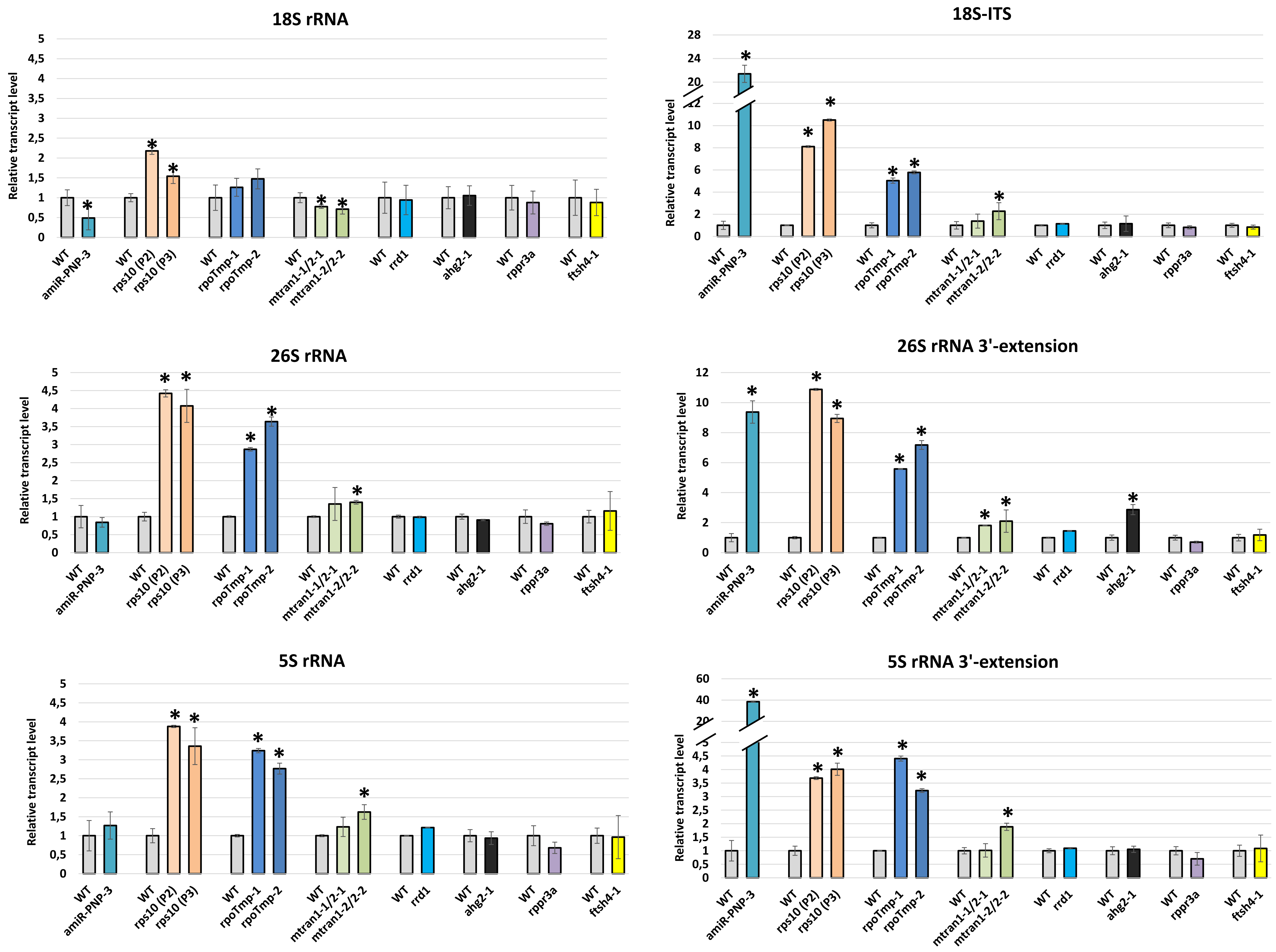

Supplemental Figure S5. RT-qPCR analysis of 18S rRNA, 26S and 5S rRNA, and their 3' extensions in mitochondrial mutants. Transcript levels are shown relative to wild-type (set to 1). Data represent means of at least three biological replicates; error bars indicate standard deviation before normalization. Statistically significant differences from wild-type are indicated by asterisks (Student's t-test; $p < 0.05$). The y-axis break allows visualization of both low and high expression values.

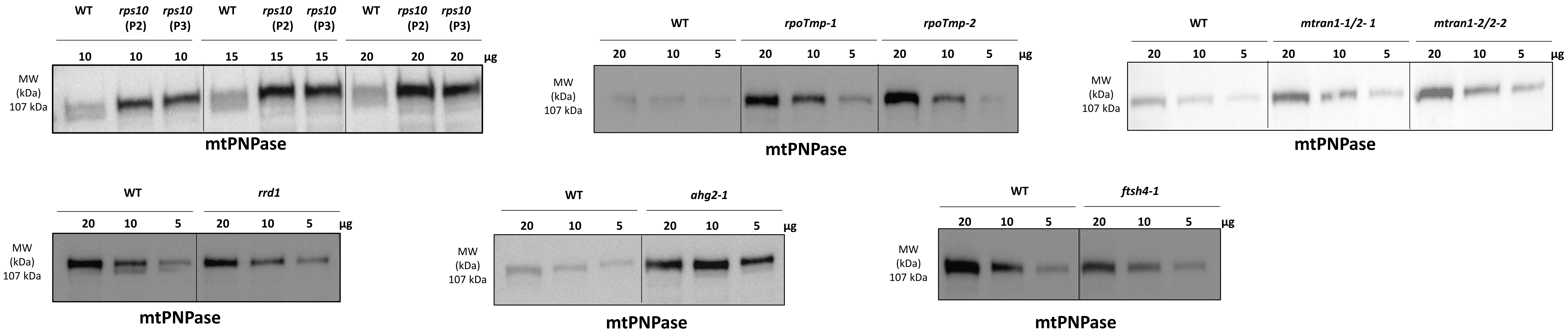

Supplemental Figure S6. Representative immunoblots of mtPNPase protein abundance in mitochondrial mutants. Serially diluted mitochondrial protein extracts were separated by SDS-PAGE, transferred to PVDF membranes, and probed with anti-mtPNPase antibodies.

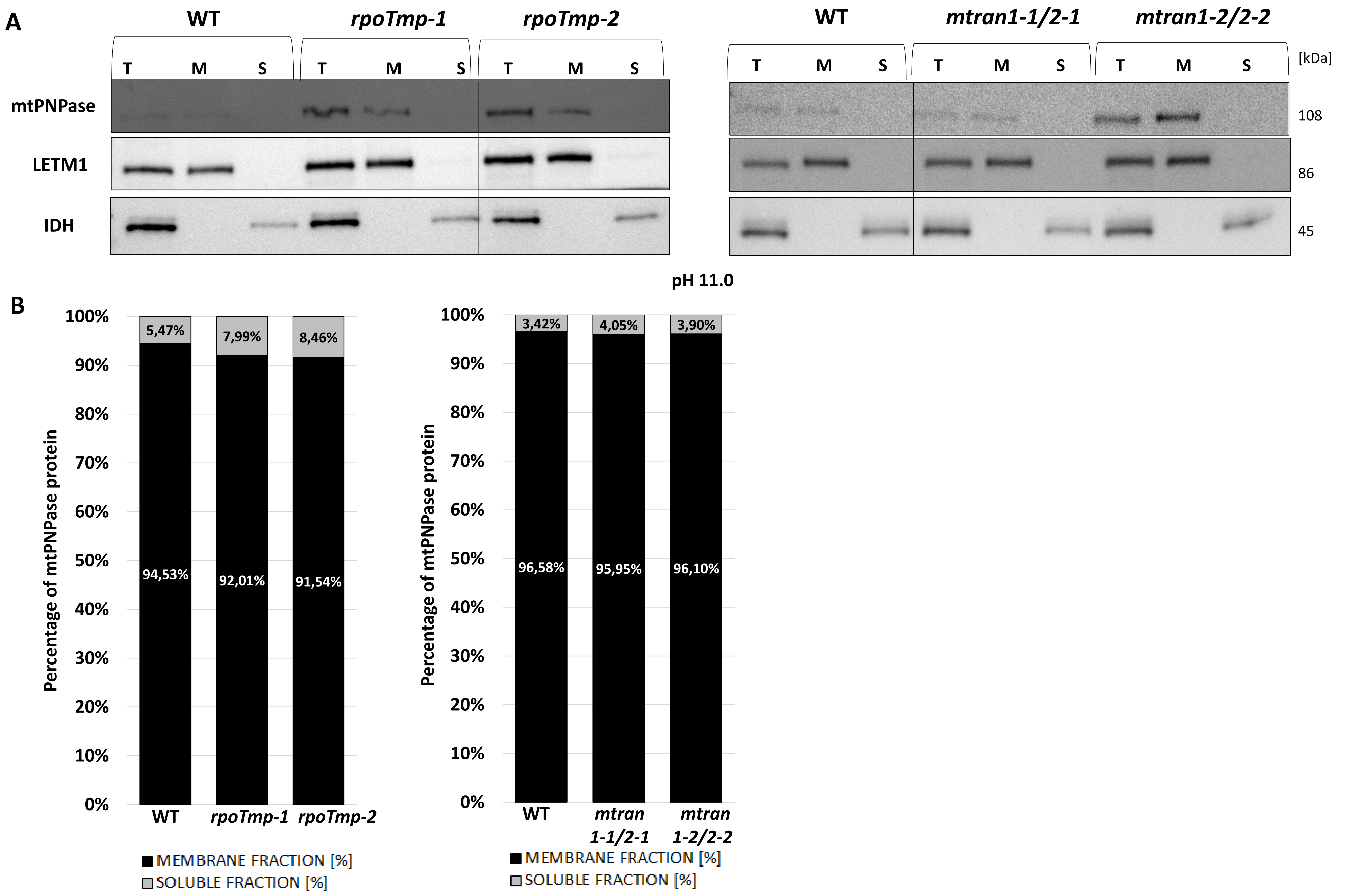

Supplemental Figure S7. Distribution of mtPNPase in membrane and soluble fractions of mitochondria in *rpoTmp-1*, *rpoTmp-2*, *mtran1-1/2-1*, and *mtran1-2/2-2*, compared with wild-type. (A) Mitochondria from WT and mutants were extracted with sodium carbonate (pH 11.0) and 50 µg of protein was separated into membrane (M) and soluble (S) fractions by centrifugation. Fractions were analyzed by SDS-PAGE and immunoblotting with antibodies against the indicated proteins. LETM1 is an integral inner membrane marker; IDH is a soluble matrix marker. T - total load of 50 µg untreated mitochondria. Molecular weight (MW) markers are shown in kDa. **(B)** Quantification of the mtPNPase amount in M and S fractions. Band intensities were measured with ImageJ and averaged for three independent experiments; values are expressed as a percentage of total protein.

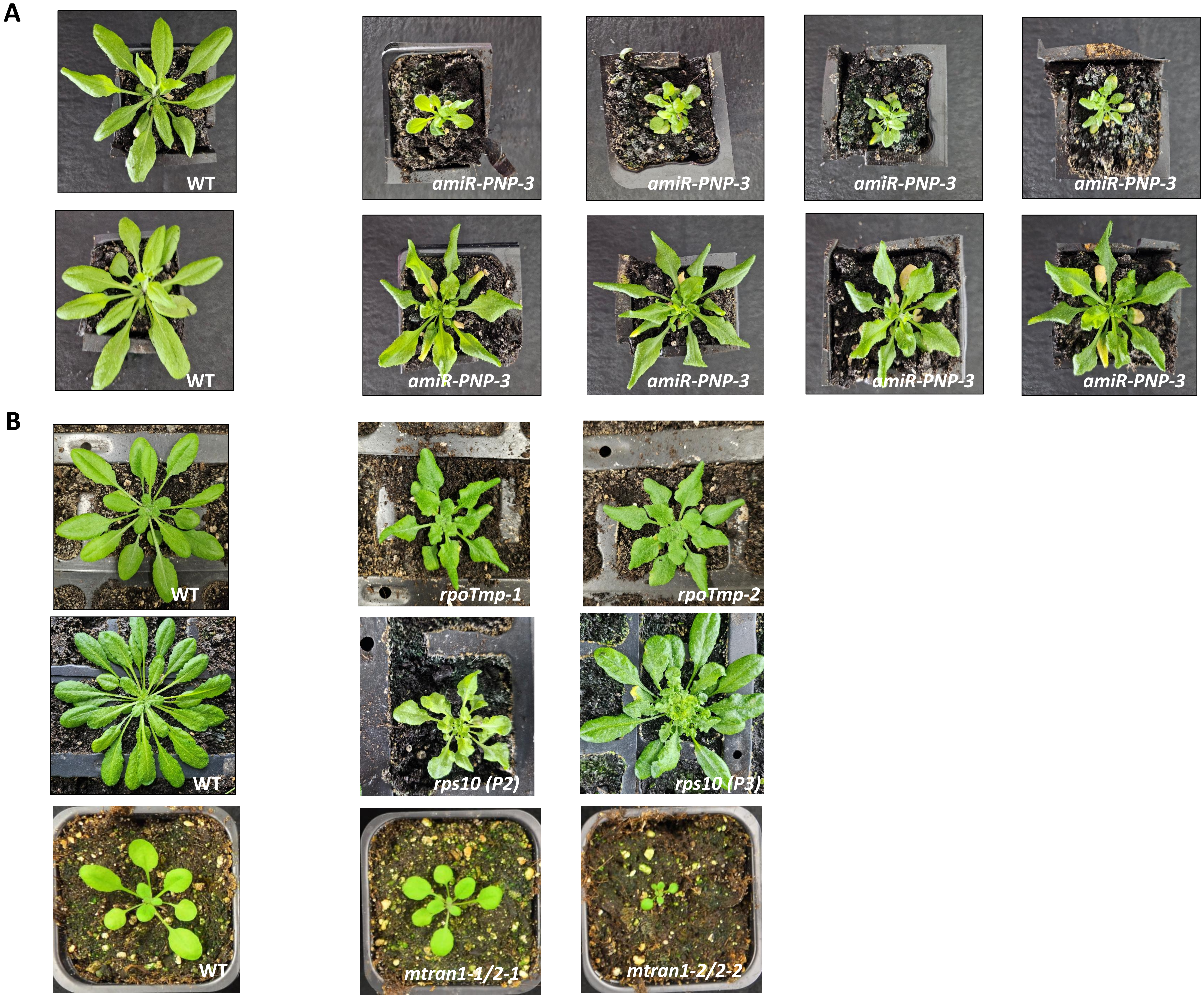

Supplemental Figure S8. Phenotypes of *amiR-PNP-3* compared with *rpoTmp, rps10* and *mtran1/2* mutants. (A) Wild-type and *amiR-PNP-3* plants grown under long-day conditions for 5–6 weeks, illustrating size variation among mutants: some are smaller (upper panel), while others are similar to wild-type (lower panel). **(B)** Wild-type and *rpoTmp* plants grown for 4.5 and 6 weeks under long-day conditions, respectively; wild-type and *rps10* plants grown for 9–10 weeks under short-day conditions; wild-type and *mtran1/2* plants grown for 25 days under long-day conditions.
